# Corynoline enhances sorafenib sensitivity in hepatocellular carcinoma via NOS3-mediated ROS production

**DOI:** 10.1186/s13020-025-01259-y

**Published:** 2025-11-14

**Authors:** Qiaoli Yi, Xi Chen, Shangjun Zhou, Jiayu Wang, Yuanliang Yan

**Affiliations:** 1 Department of Pharmacy, Xiangya Hospital, Central South University, Changsha, 410008 Hunan China; 2 Present Address: Department of Pathology, Xiangya Hospital, Central South University, Changsha, 410008 Hunan China; 3 National Clinical Research Center for Geriatric Disorders, Xiangya Hospital, Central South University, Changsha, China; 4 The Hunan Institute of Pharmacy Practice and Clinical Research, Xiangya Hospital, Central South University, Changsha, China

**Keywords:** Hepatocellular carcinoma, Sorafenib, Corynoline, NOS3, ROS

## Abstract

**Background:**

The clinical application of sorafenib (Sora) in advanced hepatocellular carcinoma (HCC) is greatly limited due to its moderate efficacy and acquired resistance. Combination therapy with other agents holds promise to improve therapeutic efficacy.

**Purpose:**

Our study aimed to screen alkaloids exerting synergistic anticancer effects with low-dose Sora in HCC treatment and underlie its molecular mechanisms.

**Methods:**

CCK-8 assay was used to evaluate the inhibition rates of Sora combined with alkaloids. The most likely binding targets were predicted by molecular docking simulations, and further verified through CETSA and DARTS. ROS levels were measured by flow cytometry. IL-18 levels were detected using ELISA. Nude mouse xenograft models were employed to validate the synergistic anticancer effect.

**Results:**

Co-administration of alkaloids, Cory showed prominent synergistic anticancer properties with Sora. Quantitative proteomic and molecular docking analyses suggested that NOS3 is a potential target of Cory. CETSA and DARTS assay revealed that Cory directly bound to NOS3. Cory increased NOS3 protein expression in a time- and concentration-dependent manner. Mechanistically, both in vitro and in vivo models showed that Cory increased the sensitivity of HCC cells to Sora through NOS3-mediated ROS production and IL-18 secretion. NOS3 knockdown could reverse the synergistic antitumor effect of Cory and Sora. The addition of antioxidant NAC reversed the increased ROS and IL-18 levels in Sora/Cory-treated Huh7 and HepG2 cells.

**Conclusions:**

This study first revealed that Cory acted synergistically with Sora to inhibit HCC growth through NOS3-mediated ROS production and IL-18 secretion, suggesting the potential of Cory as a sorafenib sensitizer.

**Supplementary Information:**

The online version contains supplementary material available at 10.1186/s13020-025-01259-y.

## Introduction

Primary liver cancer is the third most common cause of cancer-related death, following lung and colorectal cancer, and it ranked sixth in cancer incidence worldwide in 2022 [1]. The most common histological type of liver cancer is hepatocellular carcinoma (HCC), which originates from hepatocytes and accounts for 75–85% of all liver cancers [2]. The occurrence and development of HCC are closely related to chronic infections with hepatitis B virus (HBV) or hepatitis C virus (HCV), aflatoxin exposure, heavy alcohol consumption, obesity, type 2 diabetes, nonalcoholic fatty liver disease, and smoking [3–5]. The key risk factors vary from region to region. Despite multiple treatment options for HCC patients—including surgical resection, local ablation, transarterial and systemic therapies, and liver transplantation—the 5 year relative survival rates for liver cancer remain below 20% [6, 7]. Since most patients with HCC are diagnosed at an advanced stage, sorafenib (Sora), an oral multikinase inhibitor, remains an essential first-line systemic agent for the treatment of advanced HCC. The median overall survival (OS) was significantly longer in the Sora group compared to the placebo arm (10.7 months vs. 7.9 months) [8]. However, the efficacy of Sora has been dramatically challenged by the development of drug resistance within six months of initial administration [9]. Therefore, it is crucial to identify the key molecular mechanisms involved in Sora resistance to enhance its therapeutic effect in HCC.

Reactive oxygen species (ROS) are unavoidable by-products of aerobic respiration, and excessive ROS production leads to oxidative stress, causing damage to DNA, proteins, and lipids. ROS species include superoxide (O2·–), hydroxyl radicals (·OH), and hydrogen peroxide (H_2_O_2_) [10]. Cancer cells typically carry relatively high levels of ROS compared to their normal counterparts. The consequences of these increased ROS can vary significantly, with evidence indicating their dual role in facilitating and impeding malignant behavior. Increased ROS can cause DNA damage and genomic instability, thereby promoting the acquisition of oncogenic phenotypes. Conversely, ROS accumulation can limit tumor development by increasing oxidative damage and ROS-dependent death signaling [11]. Tumors often upregulate antioxidant pathways to prevent excessive oxidative damage, resulting in many tumor cells exhibiting hypersensitivity to changes in ROS levels [12]. Numerous studies have shown that compounds modulating cellular ROS levels can potentially improve the sensitivity of HCC cells to Sora [13, 14]. Pristimerin has been shown to synergistically sensitize HCC cells to Sora through ROS generation and endoplasmic reticulum stress by regulating the Akt/FoxO1/p27^kip1^ signaling pathway [15]. Tetrandrine exerted a Sora sensitization effect through ROS/Akt signaling [13]. Regulating cellular ROS may be a viable strategy to enhance Sora sensitivity in HCC.

Natural products offer ample opportunities to develop innovative medicines and are widely recognized for their medical applications [16–18]. A natural product from *Abies georgei*, named 747, has the chemical structure of kaempferol 3-(2,4-di-E-p-coumaroylrhamnoside) and exhibits high sensitivity and selectivity as a C–C motif chemokine receptor 2 (CCR2) antagonist. Compound 747 could improve the therapeutic efficacy of low-dose Sora by blocking intra-tumoral macrophage infiltration mediated by the CCL2/CCR2 axis, thereby increasing tumor-infiltrating CD8 + T cells [19]. Rhizoma Paridis saponins (RPS) are the effective products of *Paris polyphylla* (a monocot). RPS has been shown to overcome Sora intolerance and help enhance the antitumor effect of Sora in H22 tumor-bearing mice [20]. Accumulating evidence has demonstrated that alkaloid compounds such as capsaicin [21], matrine [22], tetrandrine [23], berberine [24], solamargine [25], and daurisoline [26] exhibit synergistic antitumor activity with Sora in HCC. Corynoline (Cory), a natural isoquinoline alkaloid extracted from *Corydalis bungeana* Turcz, has been reported to exhibit anti-inflammatory effects by activating the Nrf2 signaling pathway [27–30]. The antitumor effects of Cory have been reported in only a few studies [31–33]. Yi et al. showed that Cory inhibited melanoma growth in vivo and in vitro by inducing oxidative stress [32]. However, the role of Cory in HCC Sora sensitization has yet to be thoroughly investigated.

To explore the potential of alkaloids as Sora sensitizers, we utilized an alkaloid compound library and evaluated their synergistic antitumor effects in combination with Sora. Cory was screened from 269 alkaloid compounds to investigate its sensitizing effect in HCC. This study found that Cory could enhance Sora's antitumor activity by increasing cellular ROS levels both in vitro and in vivo. The results suggest that the natural product Cory may act as a potential sensitizer for Sora in the treatment of HCC.

## Materials and methods

### Cell culture and reagents

Two human HCC cell lines, HepG2 and Huh7, the normal hepatic cell line HHL5, as well as the HEK293T cell line, were obtained from the Cancer Research Institute, Central South University, China. All cell lines were cultured in Dulbecco's Modified Eagle Medium (DMEM) (Corning, 10–013-CVRC, China) supplemented with 10% fetal bovine serum (Gibco, 10091-148, USA) and 1% penicillin–streptomycin (Gibco, 15140-122, USA) at 37 °C with 5% CO2. The library of alkaloid products (269 compounds) was obtained from Selleckchem (Selleckchem, L7900, USA).

Anti-NOS3 (27120-1-AP) was obtained from Proteintech. Anti-actin (sc-58673) was purchased from Santa Cruz Biotechnology. Pierce protein A/G magnetic beads (88802) were obtained from ThermoFisher Scientific. Anti-EPHB2 (83029), anti-ubiquitin (P4D1), and rabbit (DA1E) mAb IgG antibody were obtained from Cell Signaling Technology. Anti-Ki67 (ab15580) antibody was procured from Abcam. Puromycin (S7417) was sourced from Selleck. The following reagents were used in our research: Sora (Selleck, S7397), Cory (Selleck, S9085), MG132 (Selleckchem, S2619), pronase (Roche, 10165921001), and ROS inhibitor N-acetylcysteine (NAC) (Selleck, S1623).

### Plasmids and transfection

NOS3 shRNAs (#1 5′-CCAGTACTACAGCTCCATTAA-3′, targeting CDS; #2 5′-CATCAGGAGATGGTCAACTAT-3′, targeting CDS) were purchased from Genechem (Genechem Co. Ltd., Shanghai, China). NOS3 knockdown HepG2 and Huh7 cells were generated by adding lentiviral particles produced through the transfection of HEK293T cells with the packaging plasmid psPAX2, the envelope plasmid PMD2.G, and the shRNA vector. Lipofectamine^™^ 3000 Transfection Reagent (Invitrogen, 100022052, USA) was used to co-transfect the plasmids according to the manufacturer’s instructions. After 48 h of transfection, media containing lentiviral particles were collected and used to infect cells with polybrene (MedChemExpress, HY-112735, 8 µg/ml). The cells were then harvested 48 h later and selected in puromycin (1 µg/mL) to generate a successfully transfected cell population. Huh7 and HepG2 cells were transfected with human IL18 siRNAs [IL-18 Stealth Select RNAi (HSS105406; HSS105407)] and control siRNA (Stealth RNAi™ siRNA Negative Control Kit; Invitrogen) as described previously.

### Quantitative proteomics and survival analysis

Huh7 and HepG2 cells were treated with Cory (10 µM) or DMSO control for 24 h, and the samples were collected and sent to Applied Protein Technology (Shanghai, China) for quantitative proteomics analysis. Proteomic analyses were conducted using the 4D label-free quantitative proteomic method, utilizing the timsTOF Pro ion mobility platform. Differentially expressed genes were determined based on a |log_2_fold change|≥ 1 and P < 0.05. The Xiantao academic online tools (https://www.xiantaozi.com/products) were used to generate differential gene expression heatmaps, Venn diagrams, and GO/KEGG pathway analyses. Furthermore, we evaluated the prognostic value of NOS3 expression in HCC via the HCCDB database (http://lifeome.net/database/hccdb/) [34].

### Target prediction

The canonical SMILES string for Cory was obtained from PubChem (https://pubchem.ncbi.nlm.nih.gov/compound/Corynoline#section=InChIKey) and is displayed as follows: CC12C(CC3 = CC4 = C(C = C3C1N(CC5 = C2C = CC6 = C5OCO6)C)OCO4)O. The online web server SuperPred (https://prediction.charite.de/) [35] was used to predict the potential target proteins of Cory. The UniProt ID for each protein was converted to the corresponding gene name using the UniProt Retrieve/ID mapping tool (https://www.uniprot.org/id-mapping) [36]. Molecular docking for interaction analysis was performed using MOE software (Molecular Operating Environment, version 2018.01).

### Cell counting Kit-8 assay

Cell viability was evaluated using the Cell Counting Kit-8 (CCK8) kit (Bimake, B34304, USA) according to the manufacturer's instructions. To assess the inhibition rates of Sora in combination with alkaloid compounds, Huh7 cells were inoculated in 96-well plates at a density of 2 × 10^3^ cells per well. After the cells adhered, they were treated with Sora alone (1 µM) or a drug combination (1 µM Sora and 10 µM alkaloids) for 24 h. To determine the IC_50_ value, cells were seeded in 96-well plates at a density of 2 × 10^3^ cells per well and then treated with different concentrations of Cory (0, 1, 2.5, 5, 10, 20, 40 µM) for 48 h. The cell inhibition rate was calculated using the formula: Inhibition rate = 1–(ODdrug–ODblank)/(ODcontrol–ODblank). For combination index (CI) analysis, cells were treated with a constant ratio of 10:1 Cory to Sora. The fraction affected (FA) refers to the fraction of cells that are inhibited by the drugs. CI values were calculated using Compusyn software as described by Chou [37, 38]. The drug combination effect was evaluated based on CI values: a CI value less than 1 indicates a synergistic effect, more than 1 indicates an antagonistic effect, and equal to 1 indicates an additive effect. To evaluate the role of ROS on Sora/Cory-induced cell death, cells were treated with or without ROS inhibitor NAC (5 mM) for 2 h before treatment with Sora/Cory. The OD450 was determined by a microplate reader (PerkinElmer) 2 h after 10 μL of CCK8 reagent was added.

### Colony formation assay

Cellular clonogenic potential was assessed using a colony formation assay. Cells were pretreated with Sora (1 µM) and/or Cory (10 µM) for 24 h, and then seeded in 6-well plates at a density of 2000 cells per well for 14 days. To assess the effect of ROS on Sora/Cory treatment on the colony-forming capacity of HCC cells, cells were treated with or without NAC (5 mM) for 2 h before treatment with Sora/Cory. Cell colonies were fixed and stained with 0.3% w/v crystal violet in methanol for 15–20 min, and then photographed and counted.

### Western blot

Tumor tissue samples were ground in liquid nitrogen. Cells or specimens were lysed on ice using RIPA buffer supplemented with a protease inhibitor cocktail. The supernatant was collected after centrifugation at 14,000 × g for 15 min at 4 °C. Protein concentrations were measured using the BCA protein assay kit (Thermo Fisher Scientific, 23225, USA). Denatured protein extracts (50 μg) were loaded onto 10% SDS-PAGE gels and transferred to PVDF membranes. After blocking with 5% skimmed milk at room temperature for 1 h, the membranes were incubated with primary antibodies overnight at 4 °C. Subsequently, the membranes were incubated with the appropriate secondary antibody for 1 h. For detection, the bands were visualized using Immobilon Western Chemiluminescent HRP substrate (Millipore, WBKLS0500, USA).

### ROS measurement

Intracellular ROS levels were measured using the fluorescent probe 2′,7′-dichlorofluorescein diacetate (DCFH-DA) (Sigma, 35845, USA). After the designated treatment, cells were digested with EDTA-free trypsin and washed three times with PBS. DCFH-DA, diluted to a final concentration of 10 µM, was added to the cells and incubated at 37 °C for 10 min. The fluorescence intensity of each group was assessed using a CytoFLEX (Beckman Coulter) flow cytometer at excitation and emission wavelengths of 490 and 525 nm, respectively. Gates were set based on forward scatter (FSC) vs. side scatter (SSC) plots to exclude debris and ensure the analysis of single cells. A histogram of DCFH-DA fluorescence intensity (FL1) vs. counts was created, with gates set to distinguish between fluorescence-negative and positive ROS content. The acquired data were analyzed using FlowJo software version 10.

### Apoptosis assay

Using the Annexin V-FITC apoptosis detection kit (C1062M, Beyotime), quantification of cell apoptosis was evaluated by flow cytometry (Becton Coulter). Cells were treated with vehicle, Sora (1 μM), Cory (10 μM), or Sora in combination with Cory for 72 h. After that, cells were collected, washed twice with PBS, and suspended in 195 μL binding buffer, stained with 5 μL Annexin V-FITC and 10 μL propidium iodide (PI) for 15 min in the dark. Cells were immediately subjected to flow cytometric analysis after staining. Data were processed with FlowJo 10 software, and the proportions of early-apoptotic cells (Annexin V +/PI −) and late-apoptotic cells (Annexin V +/PI +) were analyzed and plotted.

### Interleukin (IL)−18 measurement

IL-18 measurement was performed using a human IL-18 enzyme-linked immunosorbent assay (ELISA) kit (Sangon, D711091, China) according to the manufacturer’s instructions. Briefly, samples and standards were added to microplates precoated with anti-IL-18 antibody. The biotin-labeled IL-18 antibody working solution was added to the microplates for an additional 1 h incubation at 37 °C. After washing four times with elution buffer, HRP-conjugated streptavidin was applied to detect the biotin-labeled proteins. The microplates were washed to remove unbound enzyme before adding the chromogenic substrate TMB. OD values were read at 450 nm using an EnSpire 2300 Multilabel Reader (PerkinElmer, Waltham, MA, USA). A standard curve was generated by plotting the standard concentrations on the x-axis and the OD values on the y-axis to calculate the IL-18 concentration in the samples.

### Cellular thermal shift assay (CETSA)

To examine the direct binding of Cory to endogenous NOS3 in HepG2 and Huh7 cells, CETSA experiments were performed according to a previously published protocol [39]. Briefly, cells were pretreated with DMSO or Cory for 24 h, then cooled on ice and washed with PBS containing protease inhibitors. The cells were equally distributed into PCR tubes and subjected to heat shock in a Thermal Cycler (Mastercycler, Eppendorf) at gradient temperatures (37, 41, 44, 47, 50, 53, 56, 59, 63, 67 °C) for 3 min to denature proteins. Finally, cells were resuspended in NP40 buffer, subjected to three freeze–thaw cycles with liquid nitrogen to lyse the cells, and centrifuged at 20,000 × g for 20 min at 4 °C. The supernatant was collected, boiled in loading buffer, and then subjected to western blot analysis.

### Drug affinity responsive target stability (DARTS)

The DARTS assay was performed according to a previously published protocol [40]. Briefly, Huh7 and HepG2 cells were collected and total proteins were extracted using lysis buffer. Lysates were centrifuged at 14,000 × g for 20 min at 4 ℃. The supernatants were diluted 1:10 with 10 × TNC buffer (500 mM Tris–HCl, 500 mM NaCl, 100 mM CaCl2) and then treated with different concentrations of Cory or DMSO. After incubation at room temperature for 30 min, Pronase (5 μg/mL) was added to the lysates and incubated for another 30 min. Finally, the reactions were terminated by adding protease inhibitor, and the samples were analyzed by Western Blot.

### Nude mice xenograft model

Animal experiments were approved by the Institutional Animal Care and Use Committee of Central South University (Changsha, China) (Ethics Number: CSU-2023-0333). Male BALB/c nude mice (3–4 weeks old, weighing 16–18 g) were purchased from Hunan Slaccas Jingda Co., Ltd. (Changsha, China). Huh7 or Huh7-NOS3 knockdown cells (5 × 10^6^ cells per mouse) mixed with growth factor-reduced Matrigel (1:1) were subcutaneously injected into the flanks of the nude mice. Once the tumors reached approximately 50–100 mm^3^, the mice were randomly divided into four groups (n = 5), and the experiments were performed in a double-blind manner. Sora was suspended in a vehicle containing Cremophor (Selleck, S6828, USA), 95% ethanol, and sterilized ddH₂O in a 1:1:6 ratio. Cory was dissolved in a mixture of 5% DMSO, 40% PEG300, 5% Tween 80, and 50% ddH₂O. The nude mice were administered equal volumes of solvent, Sora (30 mg/kg, oral gavage, every 2 days), Cory (20 mg/kg, intraperitoneal injection, every 2 days), or a combination of Sora and Cory, according to the experimental groups. Tumor size was measured every 3 days with calipers, and tumor volume was calculated as length × width^2^ × 0.5. Serum samples were collected by centrifugation after blood samples were taken from the orbital venous plexus using capillary tubes and stored at – 80 ℃. Liver function indicators alanine aminotransferase (ALT) and aspartate transaminase (AST) were measured by ALT/AST activity assay kits (BC1555/BC1565, Solarbio, China), and kidney function indicator creatinine (Cr) was detected by Cr Assay kit (C011-2-1, Nanjing Jiancheng Biotech., China) with sarcosine oxidase method. Tumor-bearing nude mice were sacrificed by cervical dislocation on day 29 after tumor cell implantation.

### Immunohistochemistry staining

HCC tumor tissues were harvested from nude mice xenograft models. Subsequently, tissue sections underwent dewaxing, rehydration, sodium citrate-based antigen retrieval, and blockade of non-specific antibody binding with 5% normal goat serum for 20 min at room temperature. The tissue sections were then stained with primary anti-NOS3 antibody (Proteintech, 27120-1-AP, 1:50) or anti-Ki67 antibody (Abcam, ab15580, 1:200) and incubated overnight at 4 °C. Afterward, the slides were incubated with 3,3′-diaminobenzidine (DAB), counterstained with hematoxylin, and dehydrated in ethanol. Images were acquired and processed using ImageScope software (Leica Microsystems). The Ki67 proliferation index was calculated as the proportion of Ki67-positive cells to total cells (0–100%). The histologic score of NOS3 for each section was calculated using the following formula: histologic score = proportion score × intensity score. The proportion of positive cells was scored on a scale of 1 to 4 (1: 0–25%; 2: 25–50%; 3: 50–75%; 4: 75–100%). Staining intensity was graded as follows: 0 (negative), 1 (weak), 2 (moderate), and 3 (strong).

### ***H***_***2***_***O***_***2***_*** measurement***

The H₂O₂ content was measured using a hydrogen peroxide assay kit (Beyotime, S0038, China). An equal amount of tumor samples was frozen in liquid nitrogen and ground. Lysis buffer was added at a volume of 100 μL per 10 mg of tissue. After centrifugation at 12,000 × g for 5 min at 4 °C, the supernatant was collected for subsequent determination. All operations were performed on ice. Then, 50 µL of the sample and 100 µL of detection reagent were successively added to a 96-well plate. The plate was incubated at room temperature for 30 min, and the absorbance at 560 nm was measured using a microplate reader (PerkinElmer). The level of H₂O₂ in the sample was calculated according to the standard concentration curve.

### Quantitative real-time PCR

Total RNA was extracted using Trizol reagent. Then, mRNA was reverse-transcribed with SuperScript^™^ II reverse transcriptase (Invitrogen). qPCR was performed using SYBR Green PCR Master Mix (Takara). The primer sequences were as following: GAPDH forward, 5′-CAGCCTCAAGATCATCAGCA-3′, GAPDH reverse, 5′-TGTGGTCATGAGTCCTTCCA-3′; NOS3 forward, 5′-TTGGTGTTTGGCTGCCGATGC-3′, NOS3 reverse, 5′-GGTGAACCTCCGCGGCTAGC-3′; IL-18 forward, 5′-GCTTGAATCTAAATTATCAGTC-3′, IL-18 reverse, 5′- GAAGATTCAAATTGCATCTTAT-3′. Relative gene expression was calculated by 2^−ΔΔCT^ method normalized to GAPDH.

### Ubiquitination assay

The ubiquitination assay was conducted as described previously [41]. Briefly, HCC cells were treated with Cory or the control reagent. MG132 (10 μM) was applied before harvesting to prevent degradation. Then, cells were lysed with NETN lysis buffer (62.5 mM Tris-HCl pH 6.8, 20 mM NEM, 1 mM iodoacetamide, 10% glycerol, and 2% SDS). The lysates were boiled at 100 ℃ for 15 min. The reaction was diluted ten-fold with NETN buffer containing protease inhibitors, 1 mM iodoacetamide and 20 mM NEM, and then centrifuged to remove cellular debris. The supernatant was transferred and incubated with NOS3 antibody and protein A/G agarose beads sequentially at 4 °C overnight. Finally, beads were washed and subjected to immunoblotting.

### Statistical analysis

All data are presented as mean ± standard deviation (SD), and the experiments were repeated three times. Student’s t-test was applied to compare differences between two groups, while one-way ANOVA was used for comparisons involving three or more groups. Statistical analysis was performed using GraphPad Prism 8 (GraphPad Software), and P < 0.05 was considered statistically significant.

## Results

### Screening of potential alkaloids synergistic with sorafenib in HCC

**Fig. 1:**
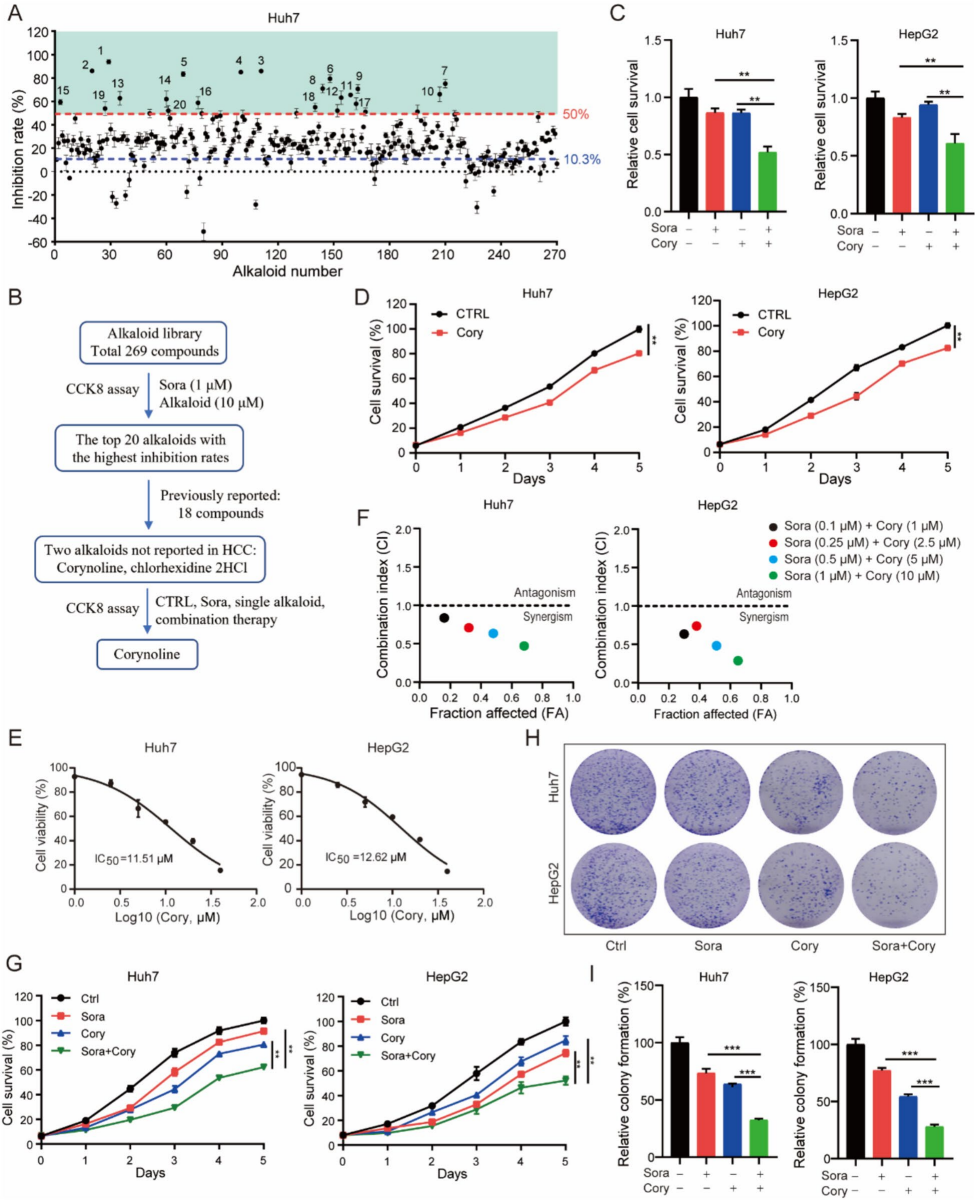
Screening of Alkaloid Compounds Synergistic with Sorafenib in HCC. **A** Scatter plot showing the combined inhibition rates of 269 alkaloid compounds (10 µM) when combined with Sora (1 µM). The x-axis represents the number of compounds, while the y-axis displays the inhibition rate. The blue dotted line indicates the inhibition rate of Sora alone (10.3%), and the red dotted line marks the 50% inhibition threshold. The top 20 alkaloids with the highest combined inhibition rates are ranked. **B** Flowchart for selecting Cory as a candidate Sora sensitizer from the alkaloid library. **C** CCK8 assay results demonstrating the effects of Cory (10 µM) and/or Sora (1 µM) for 24 h on cell viability in Huh7 and HepG2 cells. **D** CCK8 assays assessing the impact of Cory (10 µM) on the viability of the HCC cell lines Huh7 and HepG2. **E** IC_50_ values of Cory in Huh7 and HepG2 cells. **F** Combination index (CI) plot for Cory and Sora in Huh7 and HepG2 cells, with black, red, blue, and green dots representing different treatment concentrations. **G** CCK8 assays and (**H**, **I**) colony formation assays in Huh7 and HepG2 cells treated with Sora (1 µM), Cory (10 µM), or their combination. Student’s t-test was applied to compare differences between two groups, while one-way ANOVA was used for comparisons involving four groups. Statistical significance is indicated as follows: **P < 0.01, ***P < 0.001, ns: not significant

To explore the potential of alkaloid compounds as Sora sensitizers, we evaluated the inhibition rates of 269 alkaloid compounds combined with low-dose Sora (1 µM) [42, 43] in Huh7 cells (Fig. 1A, B). Table S1 presents the top 20 alkaloid compounds with the highest inhibition rates, including chlorhexidine 2HCl, (+)-fangchinoline/fangchinoline [44], sanguinarine [45, 46], fingolimod [47], sanguinarine chloride [48], dauricine [49], cepharanthine [50], chelidonine [51], daurisoline [26], dronedarone [52], vinorelbine [53], colchicine [54], sinomenine hydrochloride [55], reserpine [56], tetrandrine [23], Cory, piperlongumine [57], evodiamine [58], and tetrahydropalmatine [59], some of which have reported anti-tumor effects. To assess the antitumor effects of chlorhexidine 2HCl and Cory in Huh7 and HepG2 cells, we used the CCK8 assay. Treatment with chlorhexidine 2HCl alone significantly suppressed the proliferation of HCC cells. There was no significant difference between the combination group and the chlorhexidine 2HCl alone group (Figure S1A). However, combined treatment with Cory and Sora significantly inhibited cell proliferation compared to either Cory or Sora alone (Fig. 1C). The CCK8 assay showed that Cory significantly inhibited the proliferation of HCC cells but had no significant effects on normal hepatic cells (Fig. 1D) (Figure S1B). Based on these results, we selected Cory as a candidate Sora sensitizer for further investigation.

To determine the IC_50_ value, Huh7 and HepG2 cells were treated with different concentrations of Cory (0, 1, 2.5, 5, 10, 20, 40 µM). The results indicated that Cory suppressed the proliferation of Huh7 and HepG2 cells in a dose-dependent manner, with IC_50_ values of 11.51 µM and 12.62 µM, respectively (Fig. 1E). To assess whether the combined effects of Cory and Sora were synergistic, we calculated the combination index (CI) at various drug concentrations. The results showed that CI values at the indicated concentrations were less than 1 in both Huh7 and HepG2 cells, indicating that Cory synergizes with Sora (Fig. 1F). Furthermore, the combination treatment of Cory and Sora significantly inhibited the proliferation of HCC cells (Fig. 1G). The colony formation assay indicated that Cory synergized with Sora in inhibiting the colony-forming ability of HCC cells (Fig. 1H, I). These findings suggest that Cory could enhance the sensitivity of HCC cells to Sora.

### *Corynoline enhanced sorafenib sensitivity *in vivo

**Fig. 2:**
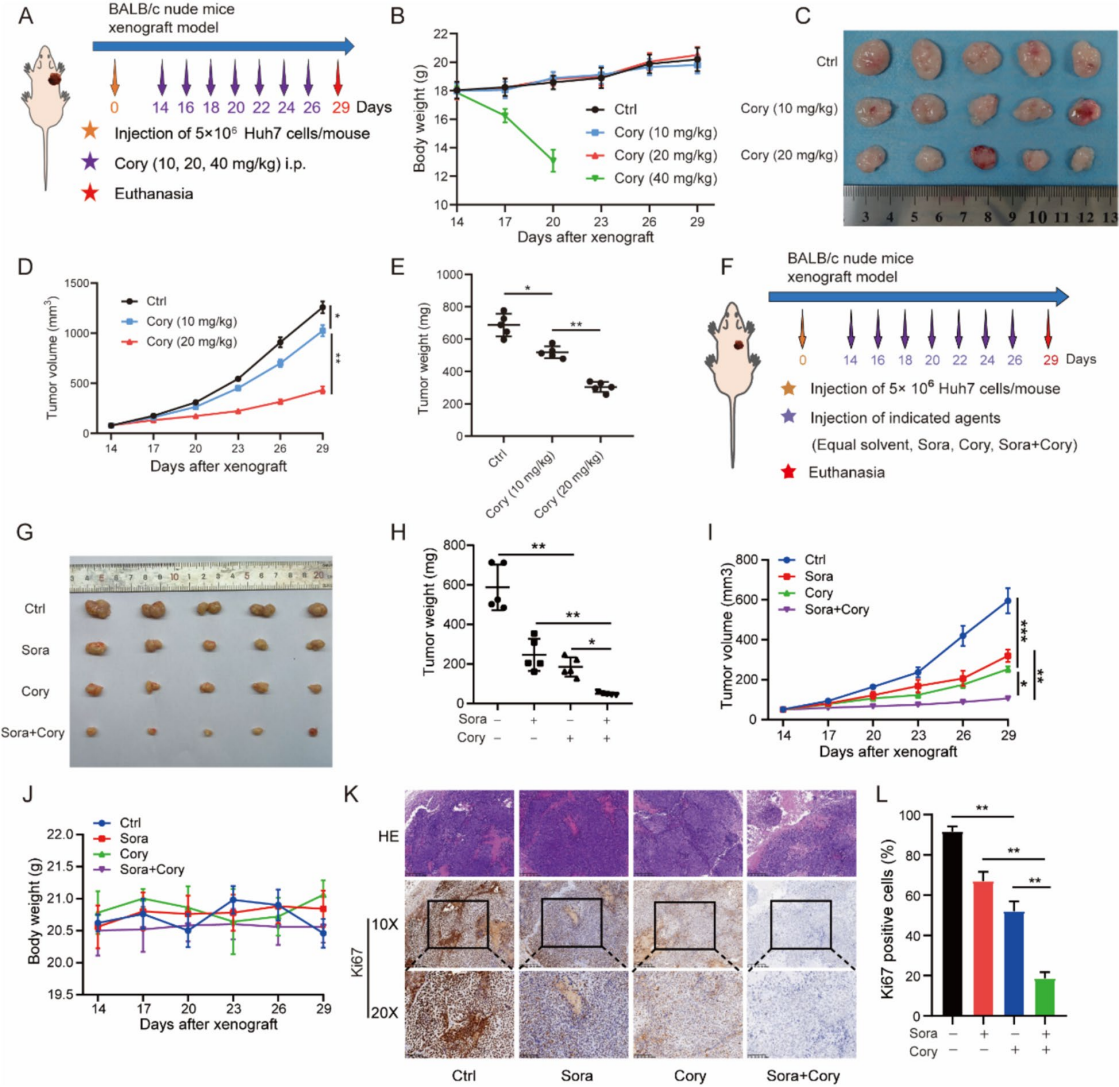
Corynoline Enhances Sorafenib Sensitivity in HCC *In Vivo*. **A** Schematic diagram for BALB/c nude mice bearing an Huh7 xenograft with either equivalent volume of solvent or Cory (10, 20, or 40 mg/kg). **B** Body weights of tumor-bearing nude mice treated with indicated dosages of Cory. **C**–**E** Tumor size (**C**), volume (**D**), and weight (**E**) of Huh7 xenografts treated with Cory were photographed and measured. **F** Schematic diagram outlining the experimental procedures for establishing the BALB/c nude mouse xenograft model. Approximately 5 × 10⁶ Huh7 cells were injected subcutaneously into the flanks of nude mice to create the tumor model. Tumor growth was monitored, and when the tumor volume reached about 50 mm^3^, mice were randomly assigned to four groups: control, Sora, Cory, and the combination group. **G** Representative images of subcutaneous tumors in each group. **H**, **I** Tumor weight and volume of mice following different treatments. **J** Body weights were monitored on specified days and presented as mean ± SD. **K**, **L** Representative images of Ki67-stained slides, along with the percentage of Ki67-positive cells in each group. One-way ANOVA was used for comparisons involving three or four groups. Statistical significance is indicated as follows: *P < 0.05, **P < 0.01, ***P < 0.001

To determine an appropriate and well-tolerated dose of Cory for in vivo study, we conducted a Huh7 tumor-bearing nude mouse model and treated mice with 10, 20, or 40 mg/kg Cory every other day (Fig. 2A). As shown in Fig. 2B, the 40 mg/kg dose led to a significant loss in body weight and was discontinued on day 20 after xenograft. In contrast, 10 and 20 mg/kg doses were well tolerated, with no significant changes in body weight throughout the experiment. Importantly, only the 20 mg/kg dose resulted in a significant inhibition of tumor growth, whereas the 10 mg/kg dose showed minimal efficacy (Fig. 2C–E). Based on these findings, we selected 20 mg/kg as an optimal dose that balances efficacy and safety in vivo. To validate the Sora-sensitizing effect of Cory in vivo, we subcutaneously implanted Huh7 cells into BALB/c nude mice and monitored tumor growth for approximately 4 weeks (Fig. 2F). As shown in Fig. 2G–I, tumor weight and volume were significantly suppressed in the nude mouse xenograft model treated with the combination of Cory and Sora compared to either treatment alone (n = 5/group). The body weight of the mice did not change significantly throughout the study (Fig. 2J). The Ki67 proliferation index was significantly downregulated in the group treated with Sora combined with Cory compared to the groups treated with Sora or Cory alone (Fig. 2K–L). H&E staining of the main organs (heart, liver, spleen, lung, and kidney) indicated that the histological morphology of the main organs was normal, with no obvious abnormalities or pathological changes (Figure S2A). In addition to histological analysis, we collected serum samples from treated mice to assess liver and kidney function by measuring alanine aminotransferase (ALT), aspartate transaminase (AST), and creatinine (Cr) levels. As shown in Figures S2B–D, treatment with Sora and/or Cory did not cause significant changes in these biochemical parameters, indicating no apparent hepatotoxicity or nephrotoxicity under our experimental conditions. These data support the conclusion that the combination of Sora and Cory has synergistic antitumor effects in vivo.

### Identification of NOS3 as the potential target for Corynoline in HCC

**Fig. 3:**
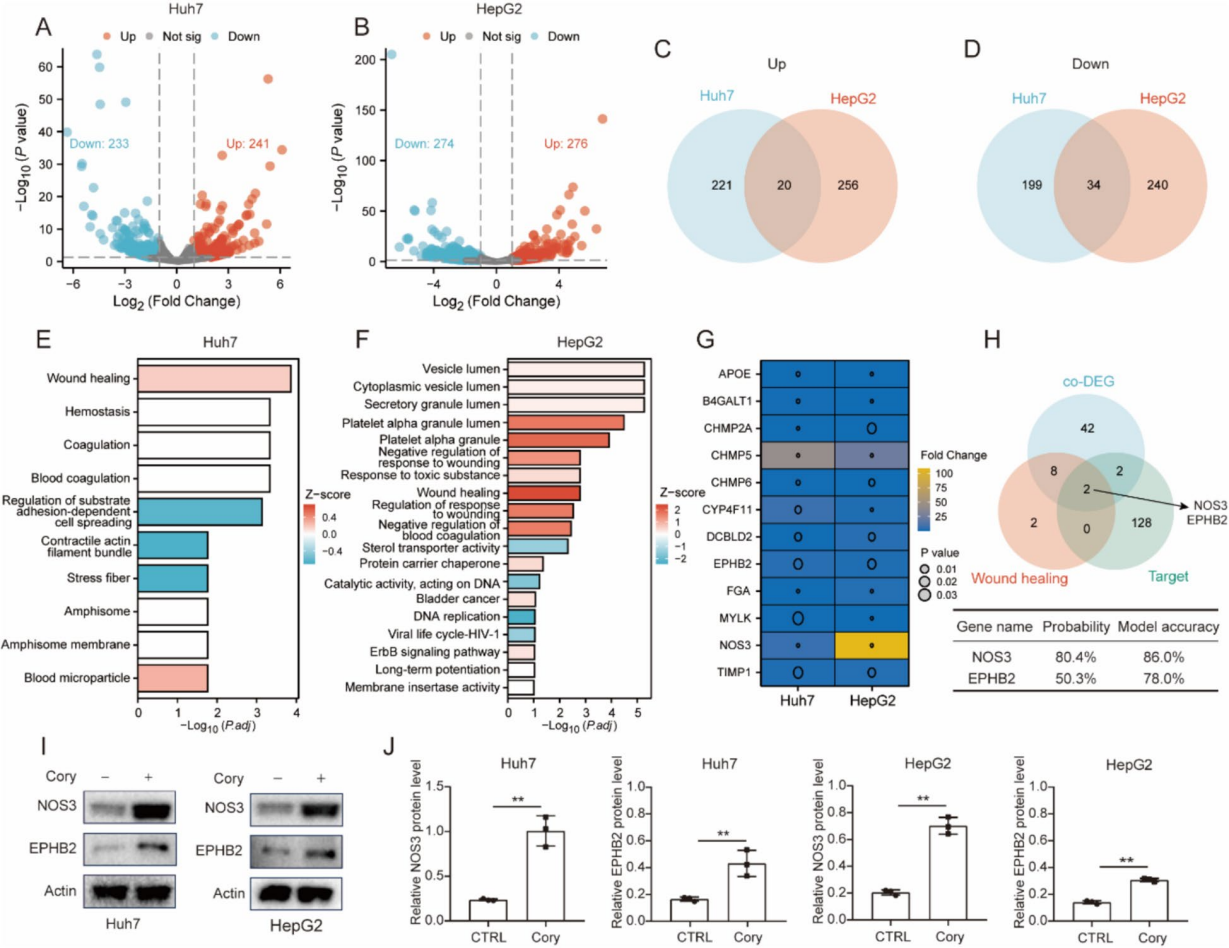
Screening of Potential Targets of Corynoline in HCC Cells. **A**, **B** Volcano plots illustrating the differential expression of proteins between the Cory treatment group and the control group. Differentially expressed proteins (DEPs) were defined as |log2fold change|≥ 1 and P < 0.05. **C**, **D** Venn diagrams depicting the overlapping differentially expressed proteins that were upregulated or downregulated in Cory-treated HCC cells. **E**, **F** Functional enrichment analysis of differentially expressed proteins in Cory-treated HCC cells. Statistically over-represented Gene Ontology terms are marked in red, while under-represented terms are shown in blue. **G** Heatmap of proteins involved in the “wound healing” pathway, with data log-transformed for improved visualization. **H** Venn diagram showing two overlapping molecules across three datasets: co-DEPs, the “wound healing” pathway, and target prediction. And the probability and model accuracy of NOS3 and EPHB2 as possible targets of Cory predicted by the SuperPred database. **I**, **J** Western blot assay showing NOS3 and EPHB2 expression levels in HCC cells treated with Cory (10 µM). Student’s t-test was applied to compare differences between two groups

To identify potential targets of Cory in HCC, we performed quantitative proteomics on Huh7 and HepG2 cells treated with Cory or DMSO. Differentially expressed proteins (DEPs) were defined as |log_2_fold change|≥ 1 and P < 0.05. The results for Huh7 and HepG2 cells are shown in Fig. 3A and B, respectively. Venn diagram analysis revealed that 20 proteins were significantly co-upregulated, while 34 proteins were co-downregulated in both HCC cell lines (Fig. 3C, D and Table S2). Functional enrichment analysis of the DEPs indicated that the “wound healing” pathway was over-represented in Cory-treated HCC cells (Fig. 3E, F). The heatmap displayed the expression levels of proteins involved in this pathway (Fig. 3G). We used the SuperPred database, which is based on a machine learning model [35], to predict potential targets of Cory (Table S3). Subsequently, we compared these predicted targets with the differentially expressed proteins (DEPs) identified within the wound healing pathway. The results indicate that NOS3 (nitric oxide synthase 3) and EPHB2 (EPH receptor B2) are possible targets for Cory, with NOS3 showing a higher probability (80.4% vs. 50.3%) and model accuracy (86% vs. 78%) compared to EPHB2 (Fig. 3H). Next, we determined the effect of Cory treatment on the expression of NOS3 and EPHB2 in HCC cells by Western Blot assay. The results showed that Cory treatment significantly enhanced the expression of NOS3 in Huh7 and HepG2 cells, with a greater effect on NOS3 compared to EPHB2 (Fig. 3I, J). Moreover, we have conducted a prognosis analysis of NOS3 and EPHB2. The results showed that higher NOS3 expression was significantly associated with a better prognosis in HCC (Figure S3A), whereas EPHB2 expression showed no association with survival (Figure S3B). Consequently, we selected NOS3 for further investigation and employed MOE software to perform a molecular docking simulation of the interaction between Cory and NOS3. Compared to positive compounds (chlorzoxazone, 3-bromo-7-nitroindazole, 5-nitroindazole, and 6-nitroindazole) [60], the in silico docking model of Cory with NOS3 resulted in a superior docking score (Figures S4A-E and Table S4), indicating that NOS3 is a potential target for Cory in HCC.

**Fig. 4:**
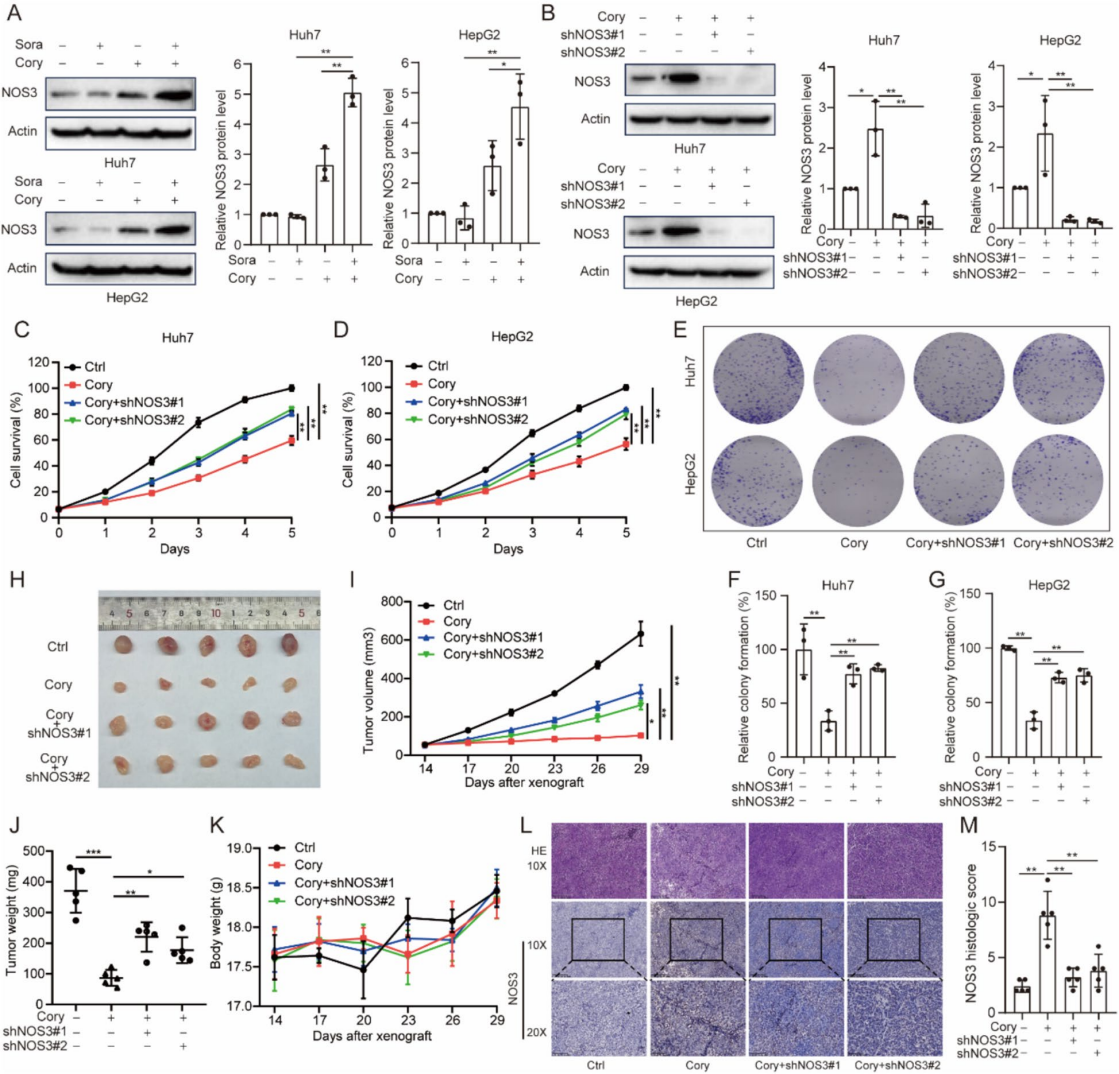
NOS3 Mediating the Antitumor Effect of Corynoline in HCC. **A** Western blot analysis showing NOS3 expression levels in indicated groups. **B** Confirmation of NOS3 knockdown in Cory-treated Huh7 and HepG2 cells via Western blot. **C**–**D** CCK8 assays and (**E**–**G**) colony formation assays assessing the impact of Cory treatment and NOS3 knockdown in HCC cells. **H** Images from the animal experiment showing subcutaneous tumors in each group. **I**, **J** Tumor weight and volume for each group, expressed as mean ± SD. **K** Monitoring of body weight in nude mice, presented as mean ± SD. **L**, **M** Representative images of IHC staining for NOS3 in xenograft tumor tissues from nude mice, along with statistical analysis of the staining. One-way ANOVA was used for comparisons involving four groups. Statistical significance is indicated as: *P < 0.05, **P < 0.01, ***P < 0.001

Furthermore, we verified the expression levels of NOS3 following treatment with Cory and/or Sora. Compared to the groups treated with a single agent, the combination group exhibited significantly elevated NOS3 protein levels in both Huh7 and HepG2 cells (Fig. 4A). Next, we detected the protein expression of NOS3 in tumor tissues treated with Sora and/or Cory by Western Blot. The results were consistent with those observed in cell culture experiments (Figure S5A). Then, we constructed stable NOS3 knockdown HCC cells. Silencing NOS3 expression with shRNAs effectively reduced NOS3 levels in Cory-treated HCC cells (Fig. 4B). CCK8 and colony formation assays demonstrated that NOS3 knockdown significantly reversed the inhibitory effect of Cory on HCC cell proliferation (Fig. 4C, G). We further evaluated the effect of NOS3 knockdown on cell survival using the CCK8 assay, and the results indicated that NOS3 knockdown alone slightly increased cell viability in Huh7 and HepG2 cells (Figure S5B). Additionally, we utilized an in vivo model to confirm these findings. In the nude mouse xenograft model, NOS3 knockdown diminished the inhibitory effect of Cory (Fig. 4H). Mice with NOS3 knockdown tumors showed increased tumor volume (Fig. 4I) and weight (Fig. 4J) when administered Cory, compared to control tumors. No significant changes in body weight were observed among the experimental groups (Fig. 4K). Moreover, IHC analysis revealed markedly reduced NOS3 protein levels in the NOS3 knockdown xenograft model, confirming the effectiveness of the knockdown (Figs. 4L, M). These results demonstrate that NOS3 is a potential target for Cory and plays an essential role in its antitumor function.

### Corynoline sensitized sorafenib through NOS3-mediated ROS production and IL-18 secretion

**Fig. 5:**
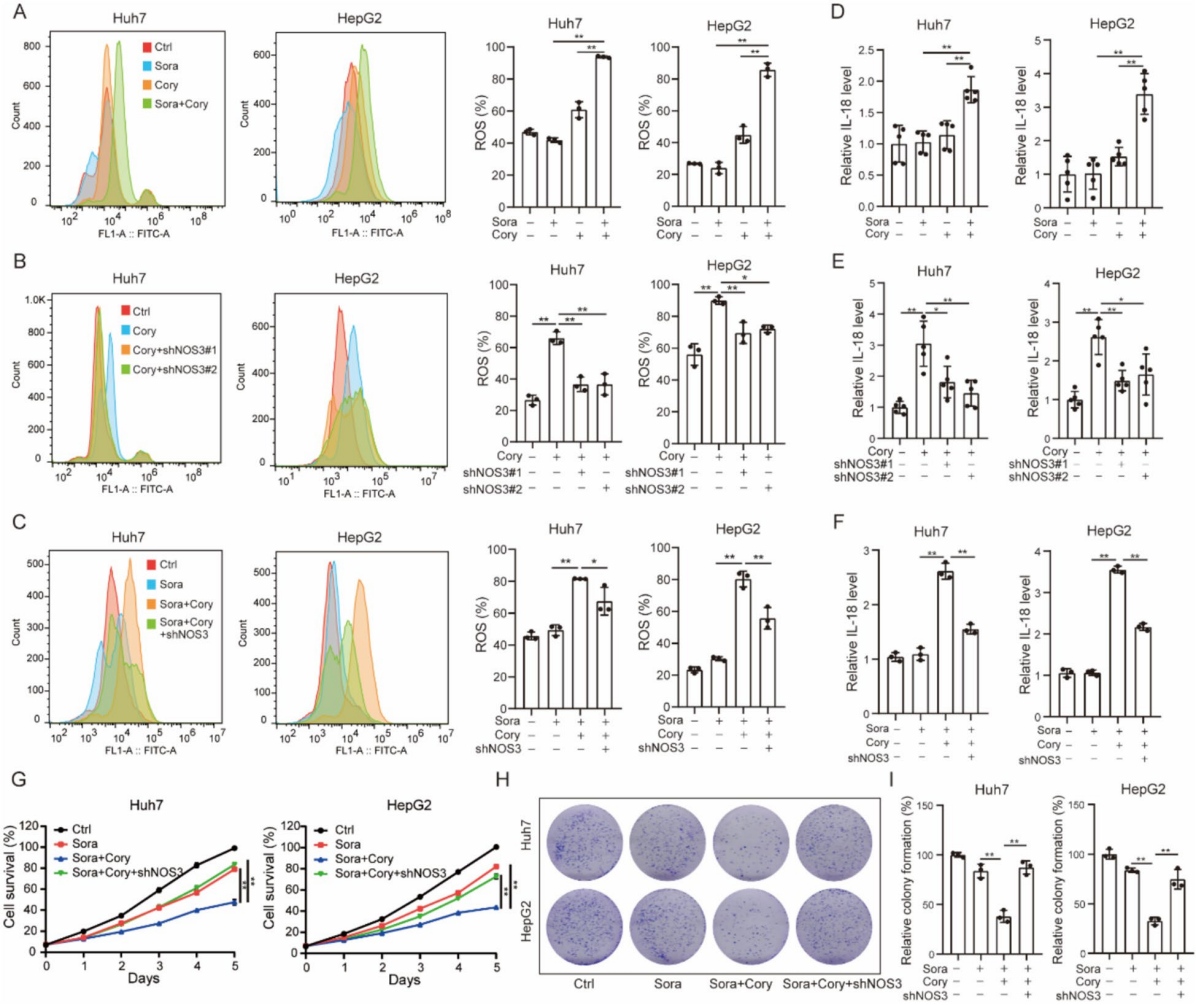
NOS3 Mediates Sorafenib Sensitivity by Regulating ROS Homeostasis and IL-18 Secretion. **A** Flow cytometric analysis of ROS levels in Huh7 and HepG2 cells treated with Cory (10 µM) and/or Sora (1 µM) for 24 h. **B** Impact of NOS3 knockdown on ROS generation in Huh7 and HepG2 cells treated with Cory (10 µM) for 24 h. **C** Effect of NOS3 knockdown on ROS generation in Huh7 and HepG2 cells treated with the combination of Cory (10 µM) and Sora (1 µM). **D** IL-18 levels measured by ELISA in HCC cells treated with Cory (10 µM) and/or Sora (1 µM) for 24 h. **E** Effect of NOS3 knockdown on IL-18 secretion in Huh7 and HepG2 cells treated with Cory (10 µM) for 24 h. **F** Effect of NOS3 knockdown on IL-18 secretion in Huh7 and HepG2 cells treated with the combination of Cory (10 µM) and Sora (1 µM). **G**, **I** Effect of combined treatment with Sora (1 µM) and Cory (10 µM), alongside NOS3 knockdown, assessed by CCK8 assays (**G**) and (**H**, **I**) colony formation assays. One-way ANOVA was used for comparisons involving three or more groups. Statistical significance is indicated as: *P < 0.05, **P < 0.01

As previously reported [61, 62], nitric oxide synthase serves as the primary source of nitric oxide (NO), which contributes to oxidative stress in conjunction with ROS. In Huh7 and HepG2 cells, we found that the combination treatment of Cory and Sora significantly increased ROS levels compared to Sora or Cory alone (Fig. 5A). Knockdown of NOS3 reversed Cory-induced ROS production (Fig. 5B). Furthermore, the knockdown of NOS3 also reversed the increase in ROS levels induced by the combination of Cory and Sora in both Huh7 and HepG2 cells (Fig. 5C). Intracellular ROS acts as secondary messengers that provoke the activation of the NLRP3 inflammasome, leading to the maturation and secretion of the pro-inflammatory cytokine IL-18 [63]. As shown in Fig. 5D, IL-18 levels were significantly higher in the combination group compared to Sora or Cory alone. NOS3 knockdown reversed the elevated IL-18 levels in Cory-treated HCC cells (Fig. 5E). Additionally, NOS3 knockdown reversed the increase in IL-18 levels induced by the combination of Cory and Sora in Huh7 and HepG2 cells (Fig. 5F). To explore the role of NOS3 in the synergistic antitumor effect of Sora and Cory, we evaluated cell proliferation using CCK8 and colony formation assays in HCC cells. As shown in Fig. 5G–I, NOS3 knockdown effectively reversed the inhibition of cell proliferation induced by the combined treatment of Cory and Sora. While NOS3 knockdown alone slightly promoted cell proliferation, its impact was more pronounced under drug treatment, where it markedly counteracted the inhibitory effects of Cory and Sora (Figure S5C). Furthermore, to elucidate the specific mechanism by which ROS contributes to Cory-mediated sensitization of HCC cells to Sora, we performed flow cytometric analysis of apoptosis using Annexin V/PI double staining. As shown in Figure S5D, the combination of Cory and Sora markedly increased apoptosis rates in Huh7 and HepG2 cells compared to treatment with either agent alone, indicating that apoptosis plays a crucial role in the observed synergistic effect.

**Fig. 6:**
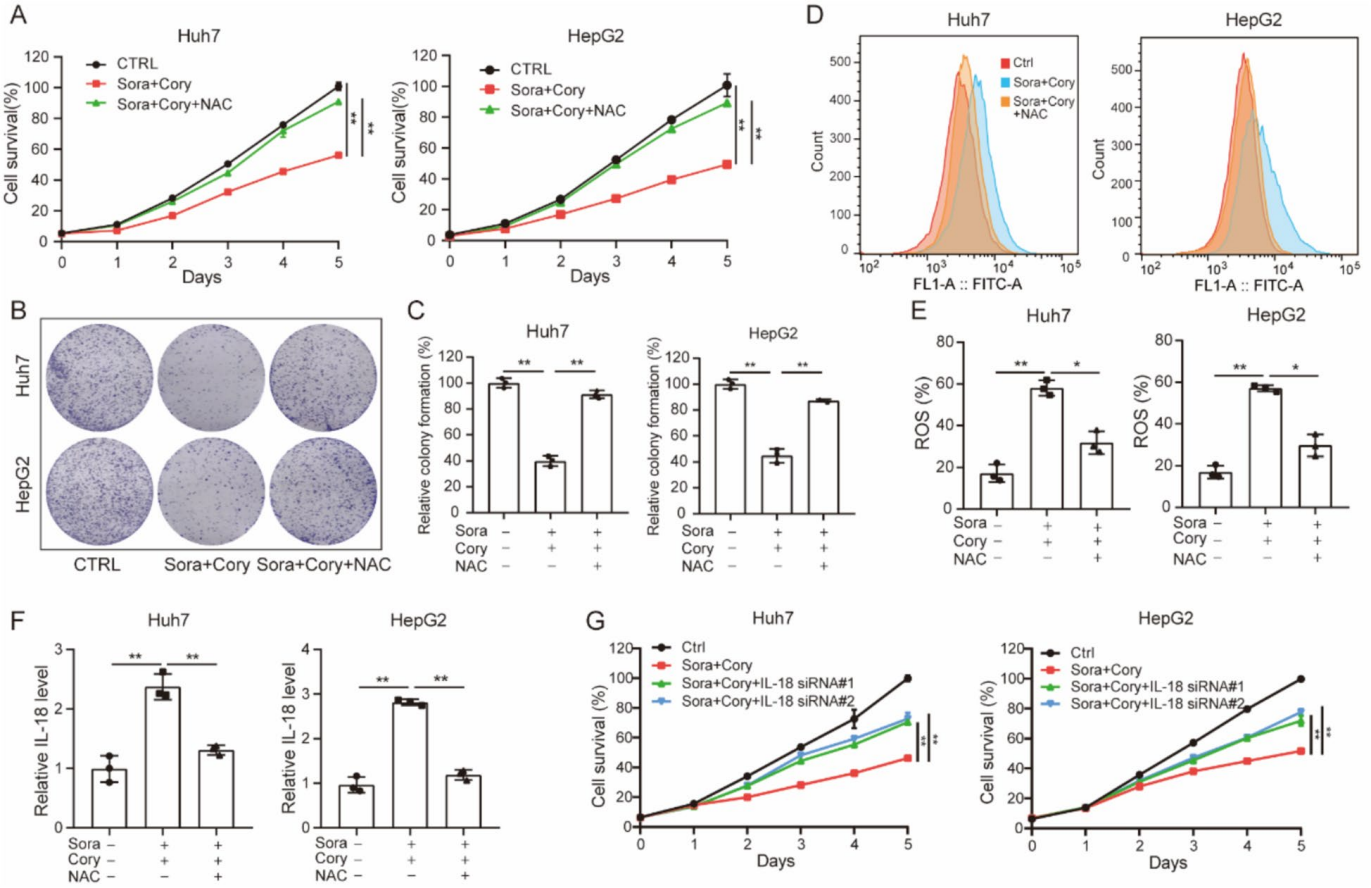
Effect of NAC and IL-18 silencing on the proliferation of Huh7 and HepG2 cells treated with Sora/Cory. **A**–**C** Effect of ROS scavenger NAC (5 mM) treatment on cell proliferation and clonal formation in Huh7 and HepG2 cells treated with Sora (1 µM)/Cory (10 µM). **D**, **E** Flow cytometry assay showing the effects of NAC on Sora/Cory-induced ROS generation. **F** ELISA assay showing the effects of NAC on Sora/Cory-induced IL-18 secretion. **G** Effect of IL-18 silencing on cell proliferation in HCC cells treated with Sora/Cory. One-way ANOVA was used for comparisons involving three or more groups. Statistical significance is indicated as: *P < 0.05, **P < 0.01

To further investigate the role of ROS in the synergistic antitumor effect of the Sora/Cory combination, Huh7 and HepG2 cells were treated with the combination, with or without the antioxidant NAC. The addition of NAC effectively reversed the inhibition of cell proliferation and colony formation induced by the Sora/Cory combination (Fig. 6A–C). Furthermore, NAC treatment reversed the increased ROS (Fig. 6D–E) and IL-18 level (Fig. 6F) in Sora/Cory-treated Huh7 and HepG2 cells. Next, to evaluate the functional relevance of IL-18 in the antitumor effect of the Cory and Sora combination, we performed siRNA-mediated knockdown of IL-18 in Huh7 and HepG2 cells. IL-18 knockdown was confirmed by qRT-PCR (Figure S5E), and CCK-8 assays demonstrated that the antiproliferative effect of the combination treatment was significantly attenuated upon IL-18 silencing (Fig. 6G). These data indicate that the combination of Cory and Sora exhibits synergistic antitumor activity through NOS3-mediated ROS production and IL-18 secretion in HCC.

### *Corynoline enhanced the *in vivo* antitumor activity of sorafenib by regulating NOS3*

**Fig. 7:**
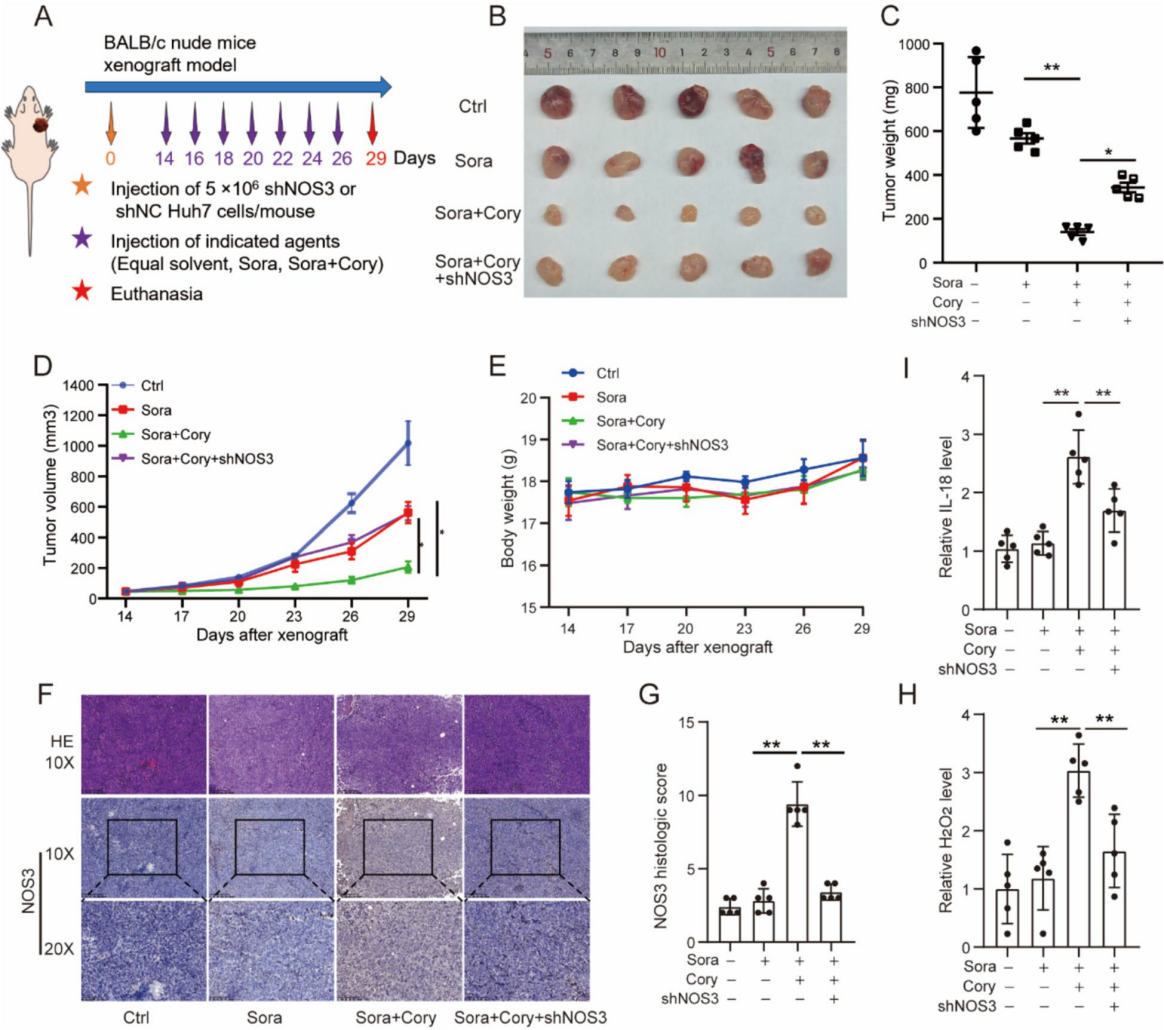
NOS3 Mediates the Synergistic Antitumor Effect of Corynoline and Sorafenib In Vivo. **A** Schematic diagram for establishing the subcutaneous xenograft tumor model in BALB/c nude mouse. Approximately 5 × 10⁶ NOS3-knocking down Huh7 cells or control Huh7 cells (shNC) were injected subcutaneously into the flanks of nude mice to create the tumor model. Tumor growth was monitored, and when the tumor volume reached about 50 mm^3^, the shNC Huh7 tumor-bearing mice were randomly divided into three groups: control, Sora, and Sora and Cory combination group. The shNOS3 Huh7 tumor-bearing mice were treated with the combined drug. **B** Images of subcutaneous tumors from each group. **C** Tumor weight and (**D**) volume for each group throughout the experiment. **E** Monitoring of body weight in mice. **F**, **G** Representative images of IHC staining for NOS3 in xenograft tumor tissues, with statistical analysis of the IHC staining results. **H**, **I** Levels of H_2_O_2_ and IL-18 in tumor tissues from the nude mouse xenograft models. One-way ANOVA was used for comparisons involving four groups. Statistical significance is indicated as: *P < 0.05, **P < 0.01, ***P < 0.001

In in vivo models, equivalent quantities of control and NOS3 knockdown Huh7 cells were subcutaneously implanted into nude mice (Fig. 7A). As expected, NOS3 knockdown diminished the synergistic antitumor effect of Cory and Sora, as evidenced by tumor weight and volume measurements (Fig. 7B–D). Throughout the experiment, no significant differences in body weight were observed between the groups (Fig. 7E). IHC staining revealed similar upregulation of NOS3 in the combination group and significant depletion of NOS3 in the knockdown group (Fig. 7F, G). Furthermore, NOS3 knockdown tumors exhibited significantly reduced levels of H_2_O_2_ and IL-18 compared to tumors treated with the combination of Cory and Sora (Fig. 7H, I). These data support the critical role of NOS3 in mediating the synergistic antitumor effect of Cory and Sora by regulating ROS homeostasis.

### Corynoline improved the stability of NOS3 protein

**Fig. 8:**
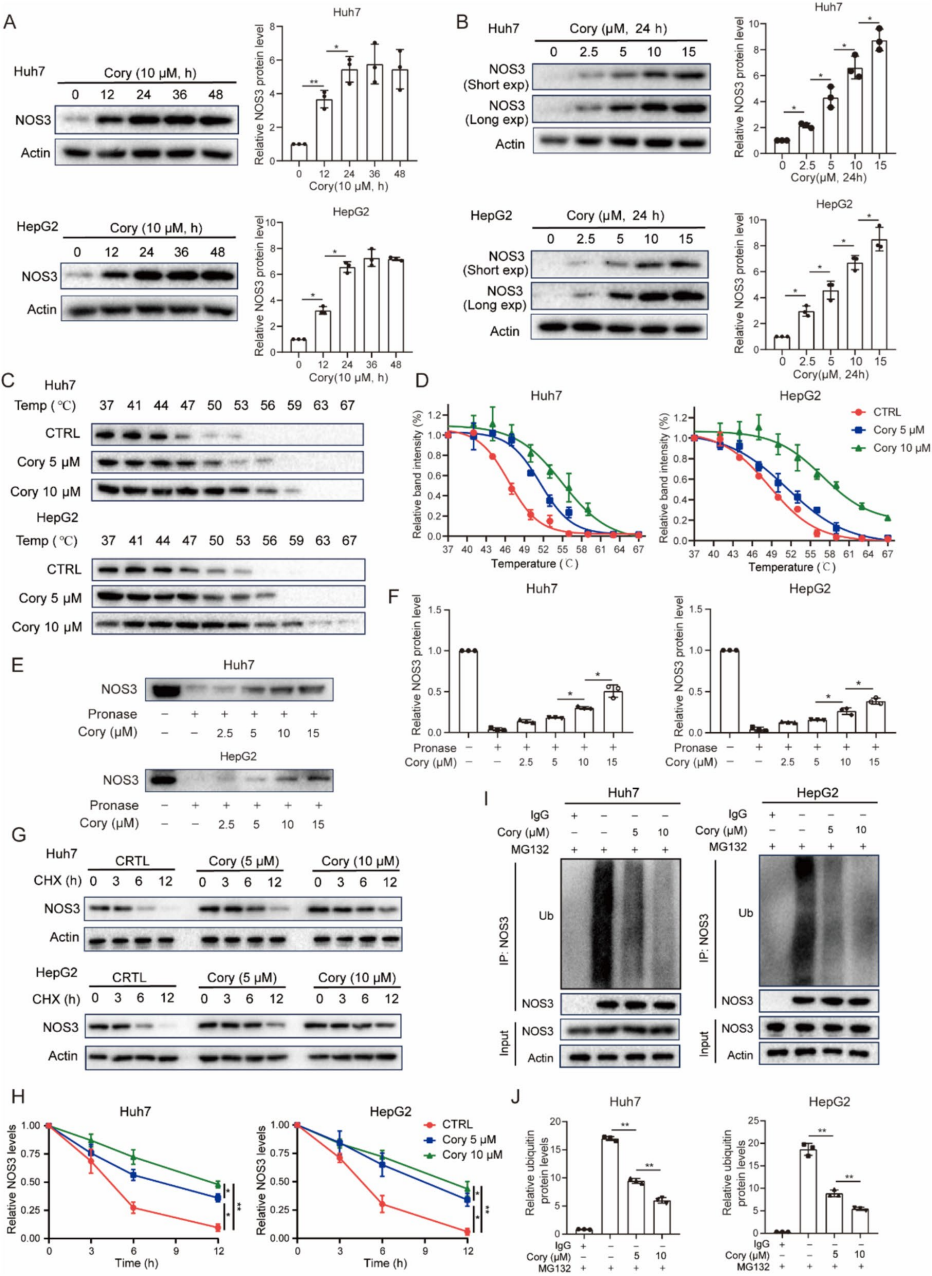
Corynoline Enhances Protein Stability of NOS3. **A**, **B** Western blot analysis of NOS3 expression in Huh7 and HepG2 cells treated with Cory at the indicated time points and concentrations. **C**, **D** CETSA assay showing that Cory promoted the resistance of NOS3 to different temperature gradients in Huh7 and HepG2 cells. **E**, **F** DARTS assay showing that Cory promoted the resistance of NOS3 to proteases in Huh7 and HepG2 cells. **G**, **H** Effects of Cory treatment on the half-life of NOS3 protein, determined by CHX chase assays. **I**, **J** In vivo ubiquitination assay indicating Cory treatment could inhibit the ubiquitination of NOS3 in Huh7 and HepG2 cells. One-way ANOVA was used for comparisons involving three or more groups. Statistical significance is indicated as: *P < 0.05, **P < 0.01

To examine the effect of Cory on the protein expression of NOS3, Huh7 and HepG2 cells were treated with Cory over various time periods. The results showed that Cory increased NOS3 protein expression in a time-dependent manner (Fig. 8A). Next, we treated HCC cells with different concentrations of Cory and found that it increased NOS3 protein expression in a concentration-dependent manner (Fig. 8B). Additionally, CETSA demonstrated that Cory directly bound to NOS3, shifting its melting curve to higher temperatures compared with DMSO, with high-dose Cory (10 μM) providing greater stabilization than low-dose (5 μM) in both Huh7 and HepG2 cells (Fig. 8C, D). Consistently, DARTS assays confirmed this direct interaction, as Cory markedly protected NOS3 from proteolysis by pronase (Fig. 8E, F). Importantly, the addition of Sora did not affect Cory-mediated stabilization of NOS3 (Figure S6), further confirming that Cory directly binds to NOS3. Quantitative RT-PCR analysis showed that NOS3 mRNA levels remained unchanged following exposure to Sora and/or Cory (Figure S5F). We then performed cycloheximide (CHX) chase assays to determine the protein stability of NOS3 in HCC cells. As illustrated in Fig. 8G, H, Cory treatment dramatically enhanced the half-life of NOS3 protein. We next determined whether Cory treatment could affect the ubiquitination of NOS3 protein by an in vivo ubiquitination assay. The results showed that Cory treatment in Huh7 and HepG2 cells significantly decreased the endogenous ubiquitination of NOS3 compared to control cells (Fig. 8I, J). These data suggest that the interaction between Cory and NOS3 inhibits the polyubiquitination of NOS3, thereby increasing its stability.

## Discussion

Sorafenib is the first approved tyrosine kinase inhibitor for treating advanced HCC; however, resistance to sorafenib limits its efficacy and availability in clinical practice. One potential way to overcome sorafenib resistance is combination therapy, such as integrating immune checkpoint inhibitors and natural products [64, 65]. Our study has revealed that Cory, a unique natural isoquinoline alkaloid, displays promising effects in attenuating the progression of HCC. Intriguingly, we observed that Cory can synergistically enhance the effects of sorafenib in HCC by regulating NOS3-mediated ROS generation and IL-18 secretion.

Although recent studies have highlighted the anti-inflammatory roles of Cory [66], its function in tumor development remains to be fully elucidated. Cory has been shown to induce cancer cell death by inhibiting Aurora kinase B activity, leading to centrosome clustering defects and mitotic arrest [67]. Yi et al. reported that Cory induces G2 arrest and apoptosis in melanoma cells via ROS generation and DNA damage, confirmed in vivo [32]. Similarly, tetrandrine and daurisoline enhance sorafenib efficacy by promoting ROS accumulation and oxidative stress-mediated apoptosis in HCC cells [13, 26]. However, their mechanisms are relatively broad and lack defined molecular targets. In contrast, Cory not only increases intracellular ROS via NOS3 activation but also directly binds to and stabilizes NOS3, as demonstrated by CETSA and DARTS in this study. Cory also exhibits favorable tissue distribution, with liver as the primary target organ [68]. Although pharmacokinetic data remain limited, the 20 mg/kg dose used here is consistent with prior studies showing good tolerability and antitumor efficacy [32, 69]. Our preliminary data showed tumor reduction without significant toxicity at this dose. These findings suggest that Cory may offer a more targeted and tolerable strategy to enhance sorafenib efficacy. However, NOS3 knockdown did not fully reverse Cory’s effects, indicating involvement of additional mechanisms. As a natural alkaloid, Cory may act through multiple targets or pathways beyond NOS3, warranting further investigation.

Sorafenib, a multi-kinase inhibitor, exerts its effects not only by suppressing tumor proliferation and angiogenesis but also by elevating intracellular ROS to promote apoptosis [70]. Given the key role of ROS in modulating cell death, tumor progression, and therapeutic resistance [71], targeting ROS pathways has become a promising strategy to enhance anti-tumor efficacy. NOS3, predominantly expressed in endothelial cells [72], normally generates nitric oxide (NO) to maintain vascular homeostasis [73, 74]. However, in the hypoxic tumor microenvironment, NOS3 can become uncoupled and produce superoxide, contributing to elevated ROS levels [61, 75]. NOS3 expression has also been observed in various tumor types [76, 77], and its function appears context-dependent, acting as either a tumor promoter or suppressor depending on cancer type and microenvironment. For instance, NOS3 is highly expressed in stomach adenocarcinoma and correlates with poor patient prognosis [78]. Conversely, some studies have shown that NOS3 overexpression can suppress tumor cell proliferation and induce cell death via increased oxidative/nitrosative stress [79]. This duality underscores the complexity of NOS3’s function in cancer and highlights the need for careful interpretation when considering its stabilization as a therapeutic strategy in HCC. In our study, NOS3 knockdown attenuated the synergistic anti-tumor effects of Cory and sorafenib, supporting a role for NOS3 in mediating drug sensitivity in HCC. While knockdown alone resulted in modest increases in cell survival in CCK8 assays, data from in vivo models were not included, which represents a limitation. As NOS3 may independently influence oxidative stress and tumor cell behavior, future studies incorporating NOS3 knockdown-only controls in long-term assays and xenograft models will be essential to distinguish its intrinsic biological effects from its contribution to drug responsiveness.

Our findings demonstrated that Cory induces NOS3 expression primarily by inhibiting its ubiquitination and enhancing protein stability. In contrast, low-dose sorafenib alone had little to no positive effect on NOS3 levels. However, combined treatment with Cory and sorafenib resulted in a more pronounced increase in NOS3 expression than Cory treatment alone, suggesting a synergistic mechanism. One possible explanation is that sorafenib influences the activity of the ubiquitin–proteasome system or regulatory kinases that control NOS3 stability. Sorafenib’s inhibition of various kinases might affect the function of E3 ubiquitin ligases or deubiquitinases, thereby altering NOS3 ubiquitination dynamics. In combination with Cory, which directly inhibits NOS3 ubiquitination, sorafenib’s modulation of upstream regulators may amplify NOS3 stabilization. This concept is supported by emerging evidence that the ubiquitination/deubiquitination balance plays a vital role in the cellular response to sorafenib in HCC [80, 81]. Future work should focus on identifying the specific ubiquitination enzymes and signaling pathways affected by sorafenib and Cory co-treatment to fully understand their cooperative impact on NOS3 regulation.

The “wound healing” pathway encompasses a series of biological processes that restore tissue integrity and function following injury, including hemostasis, inflammatory responses, ROS generation, and angiogenesis [82]. ROS can activate the NLRP3 inflammasome via TLR4/MyD88 signaling, leading to caspase-1 activation and maturation of IL-1β and IL-18 [83, 84]. Among these cytokines, IL-18 is particularly notable for its dual role: it not only promotes caspase-1-dependent pyroptosis but also enhances IFN-γ production and supports antitumor immunity via T-cell recruitment and activation [85]. Furthermore, prior studies have shown that IL-18 contributes to the therapeutic efficacy of sorafenib in HCC [86]. In our study, we identified IL-18 as a key mediator of the synergistic antitumor effect of Cory combined with sorafenib in HCC cells. IL-18 functions downstream of the ROS–NLRP3–Caspase-1 inflammasome pathway, which is activated by increased ROS production induced by Cory via NOS3 modulation. While sorafenib alone at low doses had limited effect on IL-18 secretion, its combination with Cory significantly elevated IL-18 levels, correlating with enhanced therapeutic efficacy. Although IL-1β was also upregulated, IL-18 was prioritized due to its stronger association with antitumor immune responses and sorafenib sensitivity in HCC. Future studies are warranted to explore the broader cytokine network, including the roles of IL-1β, to further clarify the immunomodulatory mechanisms underlying this combination therapy.

There are several limitations to this study. First, although molecular docking provides useful preliminary insights into the potential interaction between Cory and NOS3, it does not account for protein conformational flexibility or the complexity of the intracellular environment. The predicted binding mode may not fully reflect the actual interaction in vivo. Further validation using molecular dynamics simulations and biochemical assays is warranted [87]. Second, the lack of detailed pharmacokinetic data and comprehensive toxicity assessment limits the translational potential of Cory. Key pharmacological parameters such as bioavailability, metabolic stability, and off-target effects remain to be characterized. Third, the use of a limited number of HCC cell lines may restrict the generalizability of our findings. Future studies should include a wider panel of HCC models, including patient-derived cultures or organoids. Finally, clinical validation through large-scale, prospective studies is required to establish the safety and efficacy of Cory as a potential sorafenib sensitizer. While our findings provide novel mechanistic insights, further preclinical and clinical investigations are essential before Cory can be considered for clinical application.

## Conclusion

In conclusion, our study offers new insights into the antitumor activity of Cory against HCC and its synergistic effects when combined with sorafenib, both in vitro and in vivo. We identified NOS3 as a potential target for Cory through molecular docking analysis and CETSA. Additionally, we found that Cory enhances the sensitivity of HCC cells to sorafenib by regulating NOS3-mediated reactive oxygen species (ROS) homeostasis and IL-18 secretion. Overall, our findings suggest that the natural product Cory could serve as a promising therapeutic agent to enhance sorafenib sensitivity in HCC treatment.

## Supplementary Information

Supplementary Material 1

Supplementary Material 2: Figure S1. Cell Viability Assay of Corynoline and Chlorhexidine 2HCl. **A** CCK8 assay demonstrating the effects of chlorhexidine 2HCl and/or Sora for 24 h on cell viability in Huh7 and HepG2 cells. One-way ANOVA was used for comparisons involving four groups. **B** CCK8 assays assessing the impact of Cory on the viability of the normal hepatic cell line HHL5. Student’s t-test was applied to compare differences between two groups. Statistical significance is indicated as: **P < 0.01, ns, no significant difference

Supplementary Material 3: Figure S2. Biosafety of Corynoline and Sorafenib. **A** Representative H&E staining images of the main organsfrom nude mice, shown at magnifications of 10× and 20×. **B, C** The activity of alanine transaminase (ALT) and aspartate transaminase (AST) enzymes in the serum of nude mice was evaluated by assay kit. **D** Measurement of serum creatinine (CR). All values were within normal range. ns indicated no significant difference

Supplementary Material 4: Figure S3. Effect of NOS3 and EPHB2 Expression on HCC Patient Survival Probability. **A** Kaplan–Meier survival analysis of NOS3 expression in HCC patients using the HCCDB database. **B** Overall survival analysis of EPHB2 expression in HCC patients using the Kaplan-Meier plotter database

Supplementary Material 5: Figure S4. Molecular Docking Analysis. In silico docking models of various compounds, including Cory, chlorzoxazone, 3-bromo-7-nitroindazole, 5-nitroindazole, and 6-nitroindazole, with NOS3. The left panel displays the 3D interaction structures of the compounds with NOS3, while the right panel shows the interaction maps of these compounds with the active site amino acids of NOS3

Supplementary Material 6: Figure S5. RNA and Protein Expression of NOS3. **A** The protein levels of NOS3 in tumor tissues obtained from Cory and/or Sora-treated BALB/c nude mouse xenograft model by Western blot. **B** Effects of NOS3 knockdown on cell proliferation in Huh7 and HepG2 cells.**C** Effect of combined treatment with Soraand Cory, alongside NOS3 knockdown, assessed by colony formation assays. **D** Flow cytometric analysis of apoptosis rates in Huh7 and HepG2 cells treated with Cory and/or Sorafor 72 h. **E** Knockdown in IL-18 detected by RT-qPCR. **F** Transcriptional mRNA levels of NOS3 were analyzed in Huh7 and HepG2 cells treated with Sora and/or Cory by qPCR. One-way ANOVA was used for comparisons involving three or more groups. Statistical significance is indicated as: *P < 0.05, **P < 0.01, ns, no significant difference

Supplementary Material 7: Figure S6. The Effect of Sorafenib on NOS3 Stability in HCC Cells Treated with Corynoline. **A, B** CETSA assay showing that Cory stabilized NOS3 in Huh7 and HepG2 cells, and that this stabilization was not affected by the addition of Sora. **C, D** DARTS assay showing that Cory protected NOS3 from protease digestion in Huh7 and HepG2 cells, with Sora having no additional effect. Student’s t-test was applied to compare differences between two groups. ns is indicated as no significant difference in Cory-treated HCC cells with or without Sora

Supplementary Material 8

Supplementary Material 9

Supplementary Material 10

## Data Availability

The datasets used and/or analyzed during the current study are available from the corresponding author on reasonable request.

## Abbreviations

ALT: Alanine aminotransferase
AST: Aspartate transaminase
CETSA: Cellular thermal shift assay
CHX: cycloheximide
Cory: Corynoline
DARTS: Drug affinity responsive target stability
DEP: Differentially expressed protein
EPHB2: EPH receptor B2
HBV: Hepatitis B virus
HCC: Hepatocellular carcinoma
HCV: Hepatitis C virus
H2O2: Hydrogen peroxide
NAC: N-acetylcysteine
NOS3: Nitric oxide synthase 3
OS: Overall survival
ROS: Reactive oxygen species
Sora: Sorafenib
