# Supplementary material for: Corynoline enhances sorafenib sensitivity in hepatocellular carcinoma via NOS3-mediated ROS production
Source: Chin Med. 2025 Nov 14;20:189. doi: 10.1186/s13020-025-01259-y (PMC12616971; doi:10.1186/s13020-025-01259-y)
Supplement: Supplementary file 1 — Supplementary Material 1 [file 13020_2025_1259_MOESM1_ESM.pdf]

Table S4. The 2D structure and docking score of the first 5 conformations of corynoline, chlorzoxazone, 3-bromo-7-nitroindazole, 5-nitroindazole, and 6-nitroindazole interacting with NOS3.

Molecular	Corynoline	2D structure	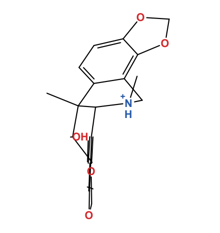	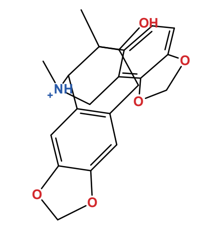	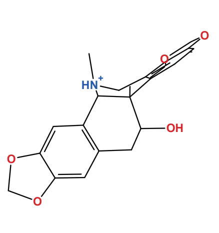	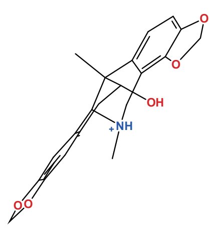	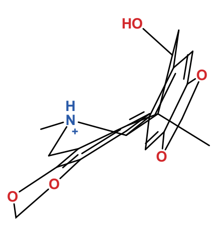
		S	-6.70	-6.54	-6.36	-6.30	-6.24
		E_score	-10.78	-11.80	-11.10	-10.56	-10.50
	Chlorzoxazone	2D structure	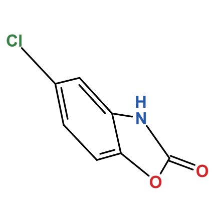	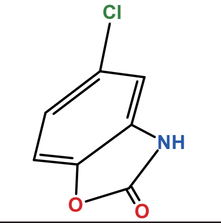	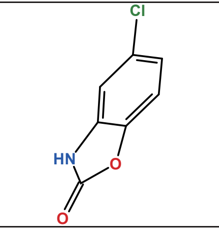	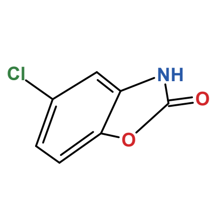	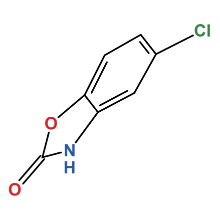
		S	-4.80	-4.68	-4.66	-4.60	-4.59
		E_score	-7.34	-7.34	-7.40	-7.98	-7.55
	3-bromo-7-nitroindazole	2D structure	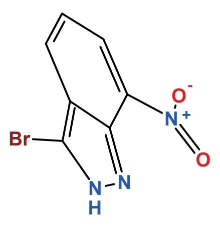	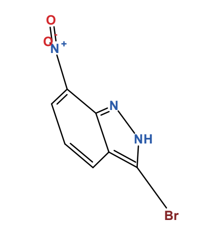	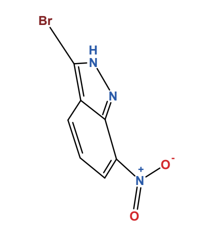	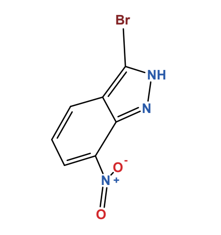	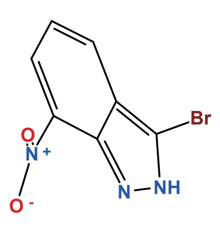
		S	-5.44	-5.34	-5.30	-4.95	-4.94
		E_score	-8.37	-8.27	-8.99	-8.37	-8.23
	5-nitroindazole	2D structure	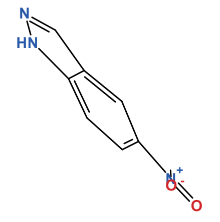	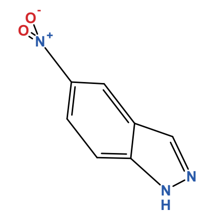	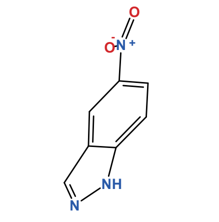	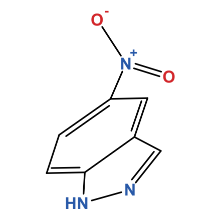	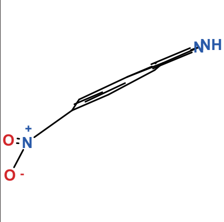
		S	-5.30	-5.20	-4.84	-4.82	-4.78
		E_score	-8.52	-8.4	-8.28	-8.30	-8.71
	6-nitroindazole	2D structure	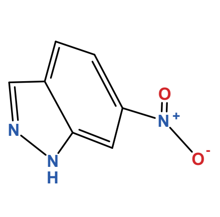	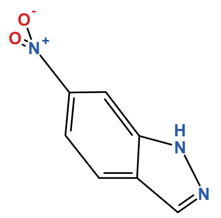	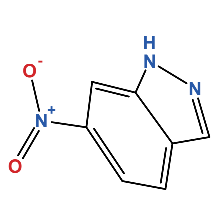	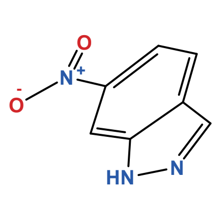	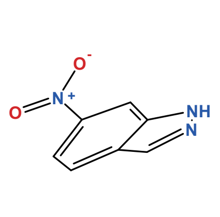
		S	-5.18	-5.15	-4.91	-4.67	-4.62
		E_score	-7.90	-8.47	-8.10	-8.13	-8.10
