# Supplementary figures and images for: Corynoline enhances sorafenib sensitivity in hepatocellular carcinoma via NOS3-mediated ROS production
Source: Chin Med. 2025 Nov 14;20:189. doi: 10.1186/s13020-025-01259-y (PMC12616971; doi:10.1186/s13020-025-01259-y)

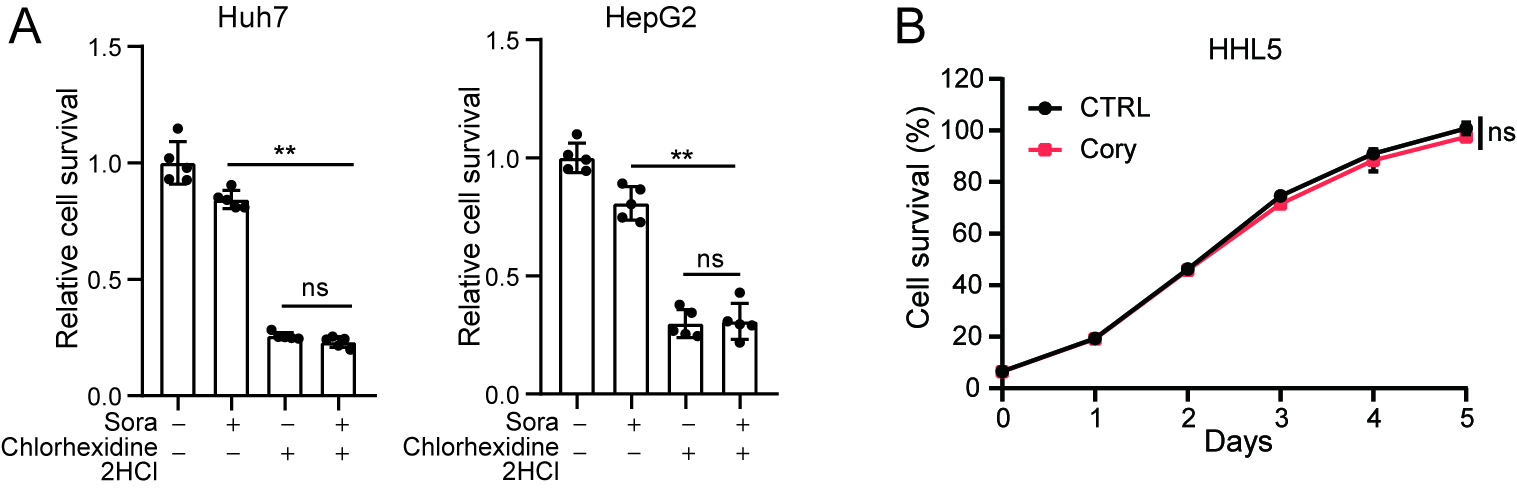

Supplement: Supplementary file 2 — Supplementary Material 2: Figure S1. Cell Viability Assay of Corynoline and Chlorhexidine 2HCl. A CCK8 assay demonstrating the effects of chlorhexidine 2HCl and/or Sora for 24 h on cell viability in Huh7 and HepG2 cells. One-way ANOVA was used for comparisons involving four groups. B CCK8 assays assessing the impact of Cory on the viability of the normal hepatic cell line HHL5. Student’s t-test was applied to compare differences between two groups. Statistical significance is indicated as: **P < 0.01, ns, no significant difference [file 13020_2025_1259_MOESM2_ESM.tif]

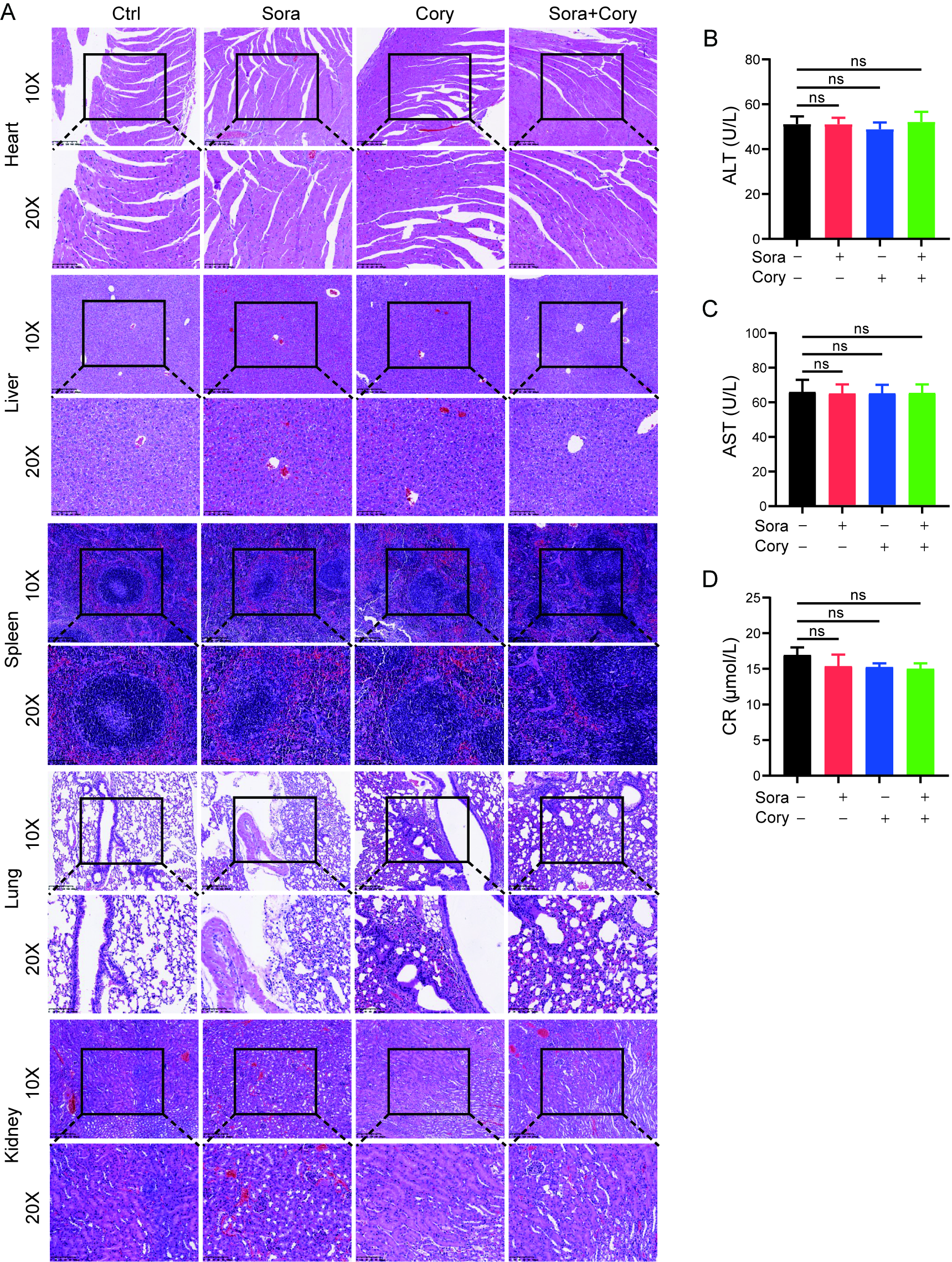

Supplement: Supplementary file 3 — Supplementary Material 3: Figure S2. Biosafety of Corynoline and Sorafenib. A Representative H&E staining images of the main organsfrom nude mice, shown at magnifications of 10× and 20×. B, C The activity of alanine transaminase (ALT) and aspartate transaminase (AST) enzymes in the serum of nude mice was evaluated by assay kit. D Measurement of serum creatinine (CR). All values were within normal range. ns indicated no significant difference [file 13020_2025_1259_MOESM3_ESM.tif]

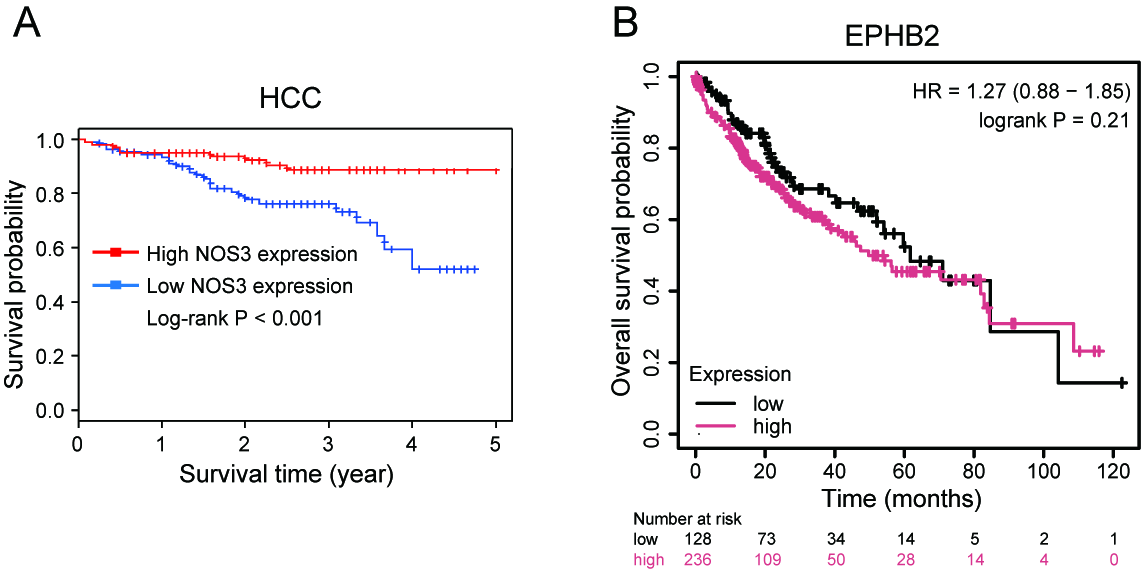

Supplement: Supplementary file 4 — Supplementary Material 4: Figure S3. Effect of NOS3 and EPHB2 Expression on HCC Patient Survival Probability. A Kaplan–Meier survival analysis of NOS3 expression in HCC patients using the HCCDB database. B Overall survival analysis of EPHB2 expression in HCC patients using the Kaplan-Meier plotter database [file 13020_2025_1259_MOESM4_ESM.tif]

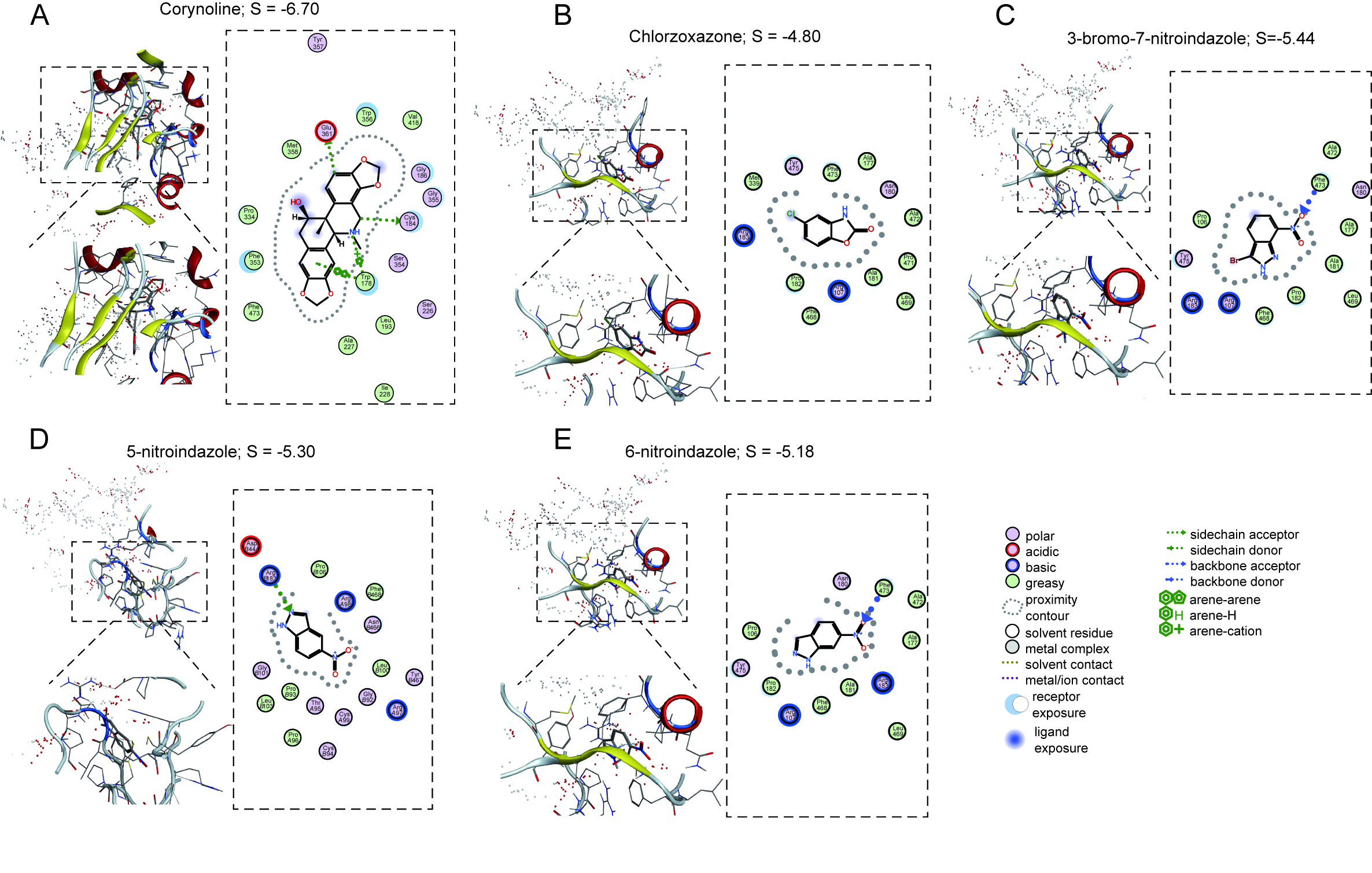

Supplement: Supplementary file 5 — Supplementary Material 5: Figure S4. Molecular Docking Analysis. In silico docking models of various compounds, including Cory, chlorzoxazone, 3-bromo-7-nitroindazole, 5-nitroindazole, and 6-nitroindazole, with NOS3. The left panel displays the 3D interaction structures of the compounds with NOS3, while the right panel shows the interaction maps of these compounds with the active site amino acids of NOS3 [file 13020_2025_1259_MOESM5_ESM.tif]

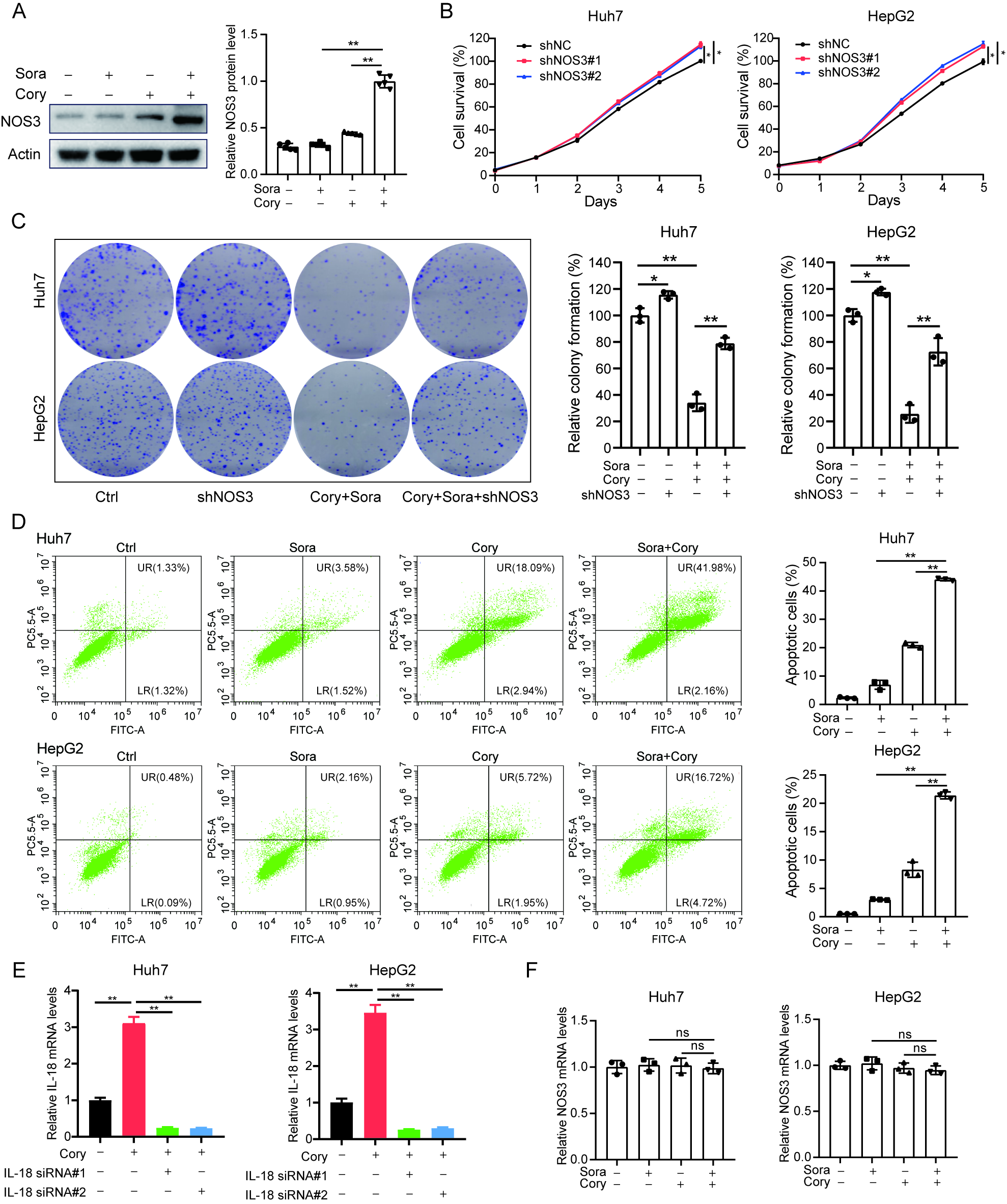

Supplement: Supplementary file 6 — Supplementary Material 6: Figure S5. RNA and Protein Expression of NOS3. A The protein levels of NOS3 in tumor tissues obtained from Cory and/or Sora-treated BALB/c nude mouse xenograft model by Western blot. B Effects of NOS3 knockdown on cell proliferation in Huh7 and HepG2 cells.C Effect of combined treatment with Soraand Cory, alongside NOS3 knockdown, assessed by colony formation assays. D Flow cytometric analysis of apoptosis rates in Huh7 and HepG2 cells treated with Cory and/or Sorafor 72 h. E Knockdown in IL-18 detected by RT-qPCR. F Transcriptional mRNA levels of NOS3 were analyzed in Huh7 and HepG2 cells treated with Sora and/or Cory by qPCR. One-way ANOVA was used for comparisons involving three or more groups. Statistical significance is indicated as: *P < 0.05, **P < 0.01, ns, no significant difference [file 13020_2025_1259_MOESM6_ESM.tif]

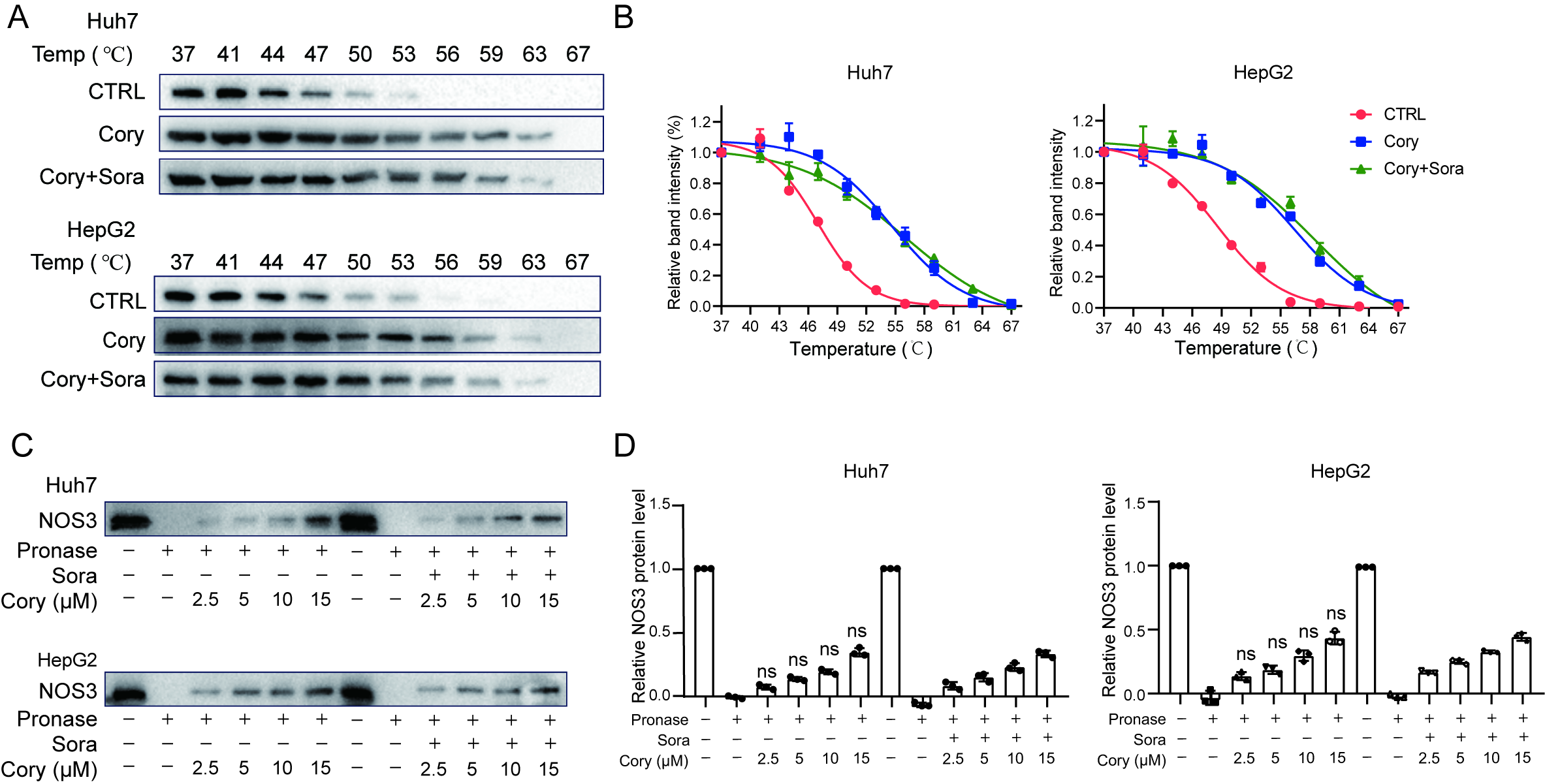

Supplement: Supplementary file 7 — Supplementary Material 7: Figure S6. The Effect of Sorafenib on NOS3 Stability in HCC Cells Treated with Corynoline. A, B CETSA assay showing that Cory stabilized NOS3 in Huh7 and HepG2 cells, and that this stabilization was not affected by the addition of Sora. C, D DARTS assay showing that Cory protected NOS3 from protease digestion in Huh7 and HepG2 cells, with Sora having no additional effect. Student’s t-test was applied to compare differences between two groups. ns is indicated as no significant difference in Cory-treated HCC cells with or without Sora [file 13020_2025_1259_MOESM7_ESM.tif]
