# Supplementary material for: Corynoline enhances sorafenib sensitivity in hepatocellular carcinoma via NOS3-mediated ROS production
Source: Chin Med. 2025 Nov 14;20:189. doi: 10.1186/s13020-025-01259-y (PMC12616971; doi:10.1186/s13020-025-01259-y)
Supplement: Supplementary file 8 — Supplementary Material 8 [file 13020_2025_1259_MOESM8_ESM.docx]

Table S1. The top 20 alkaloid compounds by combination inhibition rate with sorafenib.

| Serial number | Compound | Inhibition rate (%) | | Reports related to HCC or Sora | Ref |
| --- | --- | --- | --- | --- | --- |
| 1 | Chlorhexidine 2HCl | | 93.81 | NA | NA |
| 2 | (+)-Fangchinoline | | 86.13 | combination treatment significantly enhances Sora efficacy | ^[44]^ |
| 3 | Sanguinarine | | 85.86 | inhibiting tumor growth in HCC | ^[45, 46]^ |
| 4 | Fingolimod | | 85.06 | sensitizing HCC cells to Sora | ^[47]^ |
| 5 | Fangchinoline | | 83.44 | combination treatment significantly enhances Sora efficacy | ^[44]^ |
| 6 | Sanguinarine chloride | | 79.33 | inducing the apoptosis of HCC cells | ^[48]^ |
| 7 | Dauricine | | 75.19 | elevating the sensitivities of HCC cells to Sora | ^[49]^ |
| 8 | Cepharanthine | | 71.01 | inhibiting tumor growth in HCC | ^[50]^ |
| 9 | Chelidonine | | 70.63 | enhancing the antitumor effect of lenvatinib on HCC | ^[51]^ |
| 10 | Daurisoline | | 66.20 | enhancing sensitivity of HCC cells to Sora | ^[26]^ |
| 11 | Dronedarone | | 65.59 | inducing the apoptosis of HepG2 cells | ^[52]^ |
| 12 | Vinorelbine Tartrate | | 63.17 | inhibiting tumor growth in HCC | ^[53]^ |
| 13 | Colchicine | | 62.70 | promoting Sora anti-cancer effects on HCC | ^[54]^ |
| 14 | Sinomenine hydrochloride | | 61.94 | inhibiting tumor growth in HCC | ^[55]^ |
| 15 | Reserpine | | 59.46 | inhibiting tumor growth in HCC | ^[56]^ |
| 16 | Tetrandrine | | 58.86 | increasing the sensitivities of HCC cells to Sora | ^[23]^ |
| 17 | Corynoline | | 57.88 | NA | NA |
| 18 | Piperlongumine | | 55.09 | synergistically enhancing the antitumor effect of Sora | ^[57]^ |
| 19 | Evodiamine | | 53.87 | inhibiting tumor growth in HCC | ^[58]^ |
| 20 | Tetrahydropalmatine hydrochloride | | 52.06 | inhibiting tumor growth in HCC | ^[59]^ |
